# Supplementary material for: Identification of an Orally Bioavailable, Brain-Penetrant Compound with Selectivity for the Cannabinoid Type 2 Receptor
Source: Molecules. 2022 Jan 14;27(2):509. doi: 10.3390/molecules27020509 (PMC8778670; doi:10.3390/molecules27020509)
Supplement: Supplementary file 1 [file molecules-27-00509-s001.zip › molecules-1543300-supplementary.pdf]

# Supplementary Information



| Page Number | Figure |
|---|---|
| SI1 | $^{1}H$ NMR spectrum of 4a |
| SI2 | $^{13}C$ NMR spectrum of 4a |
| SI3 | $^{1}H$ NMR spectrum of 4b |
| SI4 | Dept-135 NMR spectrum of 4b |
| SI5 | $^{1}H$ NMR spectrum of 4c |
| SI6 | $^{13}C$ NMR spectrum of 4c |
| SI7 | $^{1}H$ NMR spectrum of 4d |
| SI8 | $^{13}C$ NMR spectrum of 4d |
| SI9 | $^{1}H$ NMR spectrum of 4e |
| SI10 | Dept-135 NMR spectrum of 4e |
| SI11 | $^{1}H$ NMR spectrum of 4f |
| SI12 | $^{13}C$ NMR spectrum of 4f |
| SI13 | $^{1}H$ NMR spectrum of 4g |
| SI14 | $^{13}C$ NMR spectrum of 4g |
| SI15 | $^{1}H$ NMR spectrum of 4h |

| Page Number | Figure |
|---|---|
| SI16 | $^{13}$C NMR spectrum of 4h |
| SI17 | $^{1}$H NMR spectrum of 4i |
| SI18 | $^{13}$C NMR spectrum of 4i |
| SI19 | $^{1}$H NMR spectrum of 4j |
| SI20 | $^{13}$C NMR spectrum of 4j |
| SI21 | $^{1}$H NMR spectrum of 4k |
| SI22 | $^{13}$C NMR spectrum of 4k |
| SI23 | $^{1}$H NMR spectrum of 4l |
| SI24 | $^{13}$C NMR spectrum of 4l |
| SI25 | $^{1}$H NMR spectrum of 4m |
| SI26 | $^{13}$C NMR spectrum of 4m |
| SI27 | $^{1}$H NMR spectrum of 4n |
| SI28 | $^{13}$C NMR spectrum of 4n |
| SI29 | $^{1}$H NMR spectrum of 4o |
| SI30 | $^{13}$C NMR spectrum of 4o |

| Page Number | Figure |
|---|---|
| SI31 | $^{1}$H NMR spectrum of 4p |
| SI32 | $^{13}$C NMR spectrum of 4p |
| SI33 | $^{1}$H NMR spectrum of 4q |
| SI34 | $^{13}$C NMR spectrum of 4q |
| SI35 | Dept-135 NMR spectrum of 4q |
| SI36 | Typical MRM chromatograms of **4k** (top panel) and the IS (below panel) at 0.5 h plasma pharmacokinetic study sample |
| SI37 | Typical MRM chromatograms of **4k** (top panel) and the IS (below panel) at 0.5 h brain pharmacokinetic study sample |
| SI38 | Typical MRM chromatograms of **4k** (top panel) and the IS (below panel) at 0.5 h kidney pharmacokinetic study sample |
| SI39 | Typical MRM chromatograms of **4k** (top panel) and the IS (below panel) at 0.5 h liver pharmacokinetic study sample |

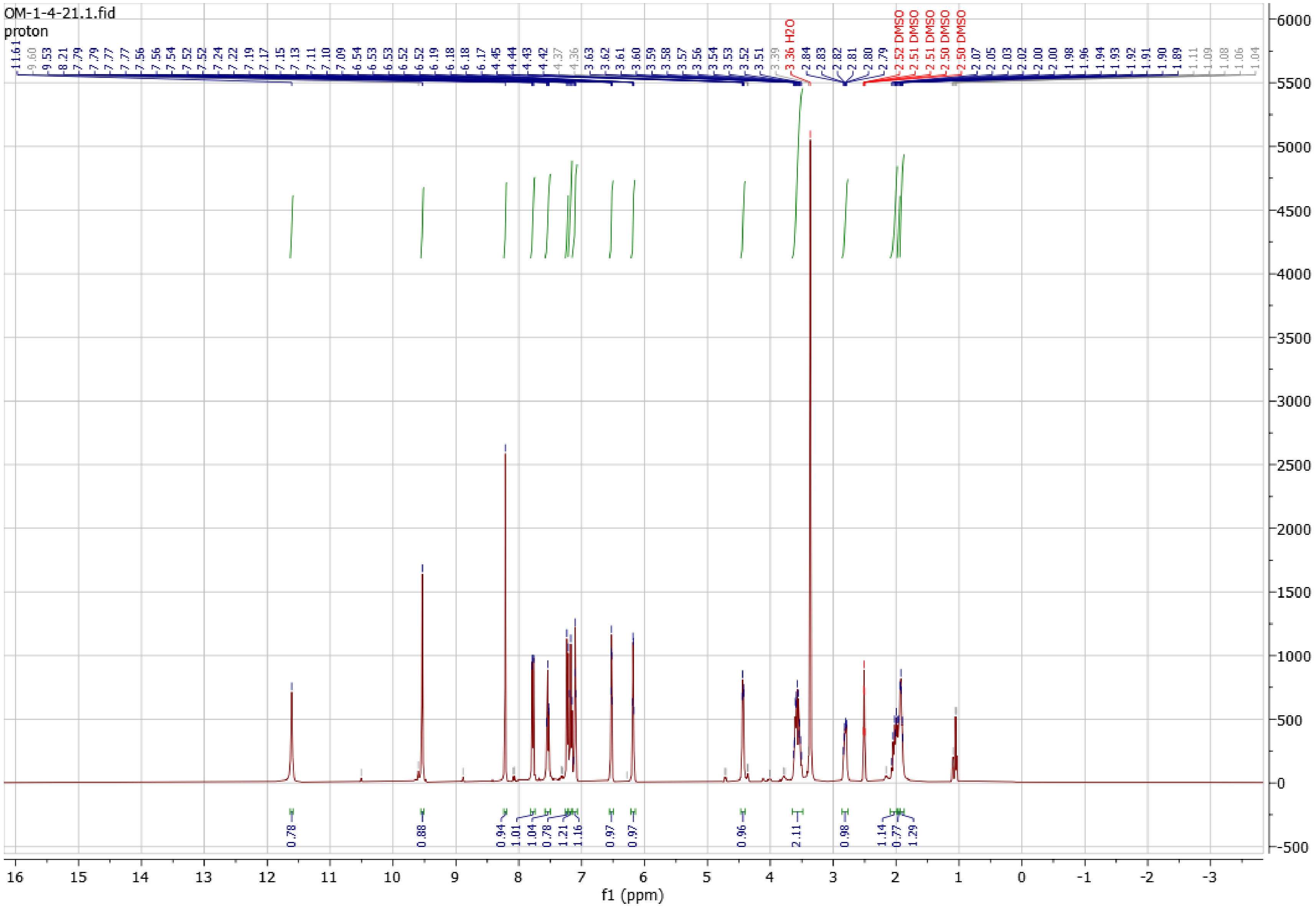

**SI 1:** $^{1}$H NMR spectrum of 4a [400 MHz,  DMSO-*d6* ]

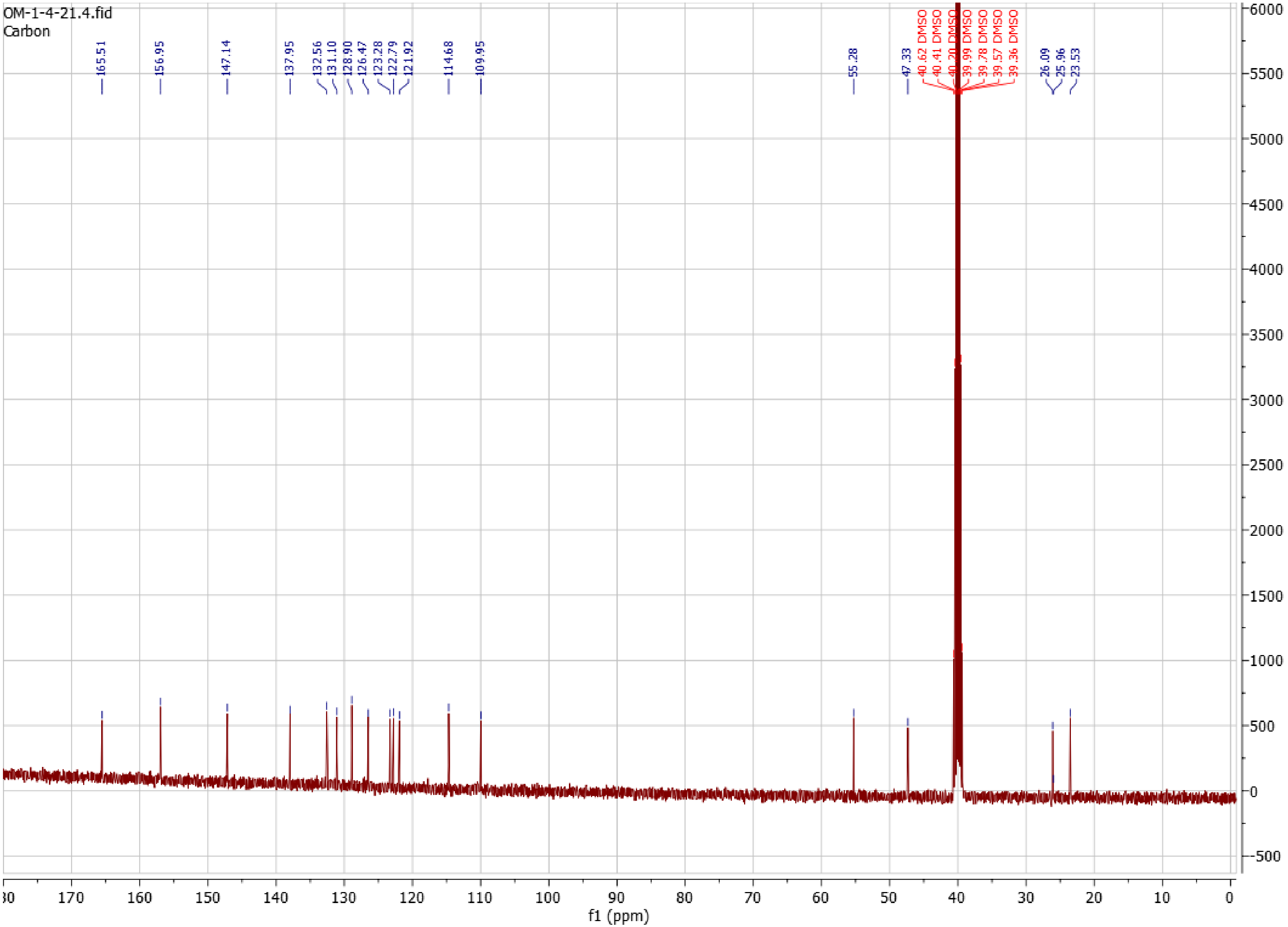


**SI 2:** $^{13}$C NMR spectrum of 4 a [100 MHz, DMSO-*d6* ]

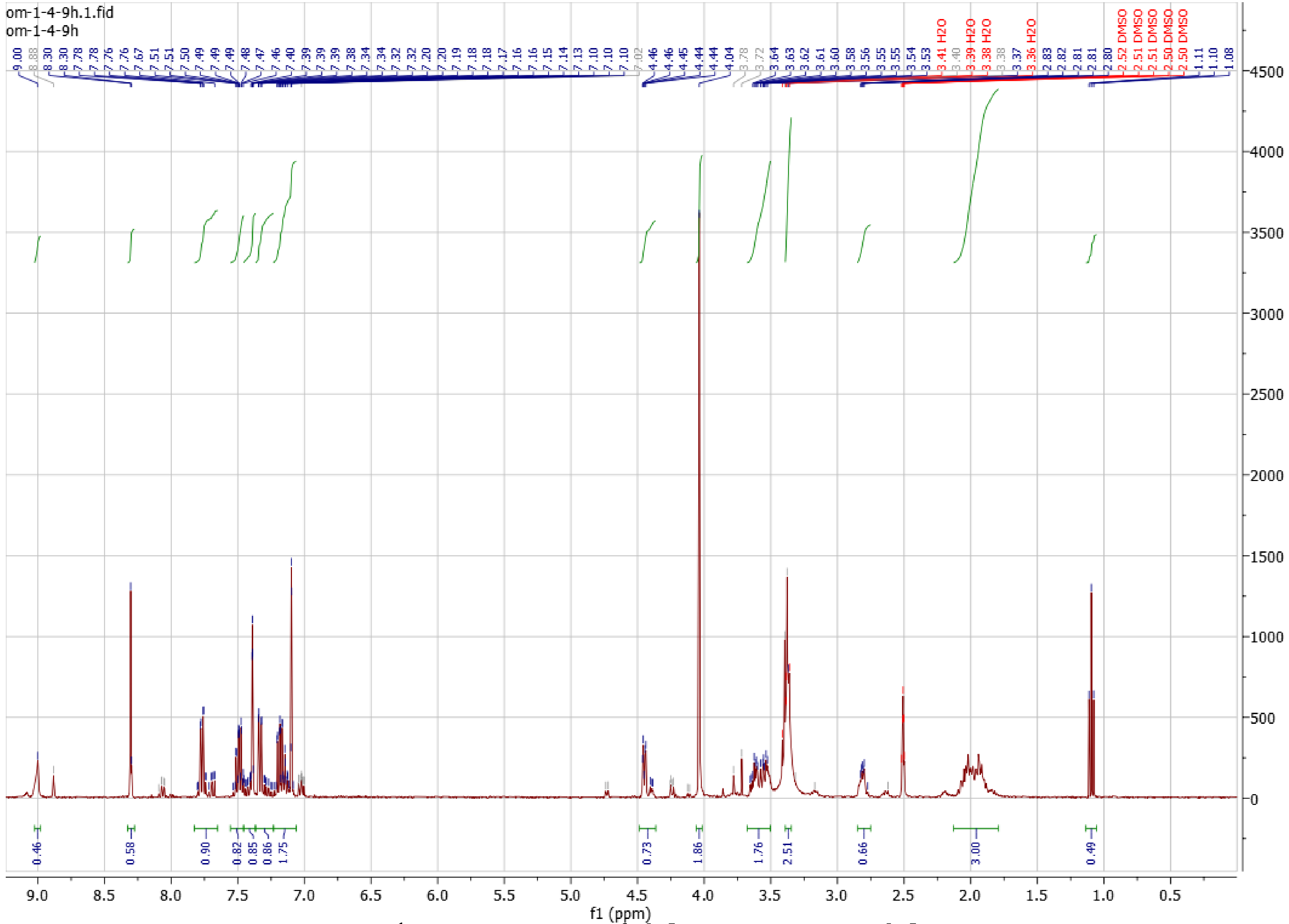


**SI 3:** $^{1}$H NMR spectrum of 4b [400 MHz, DMSO-*d6* ]

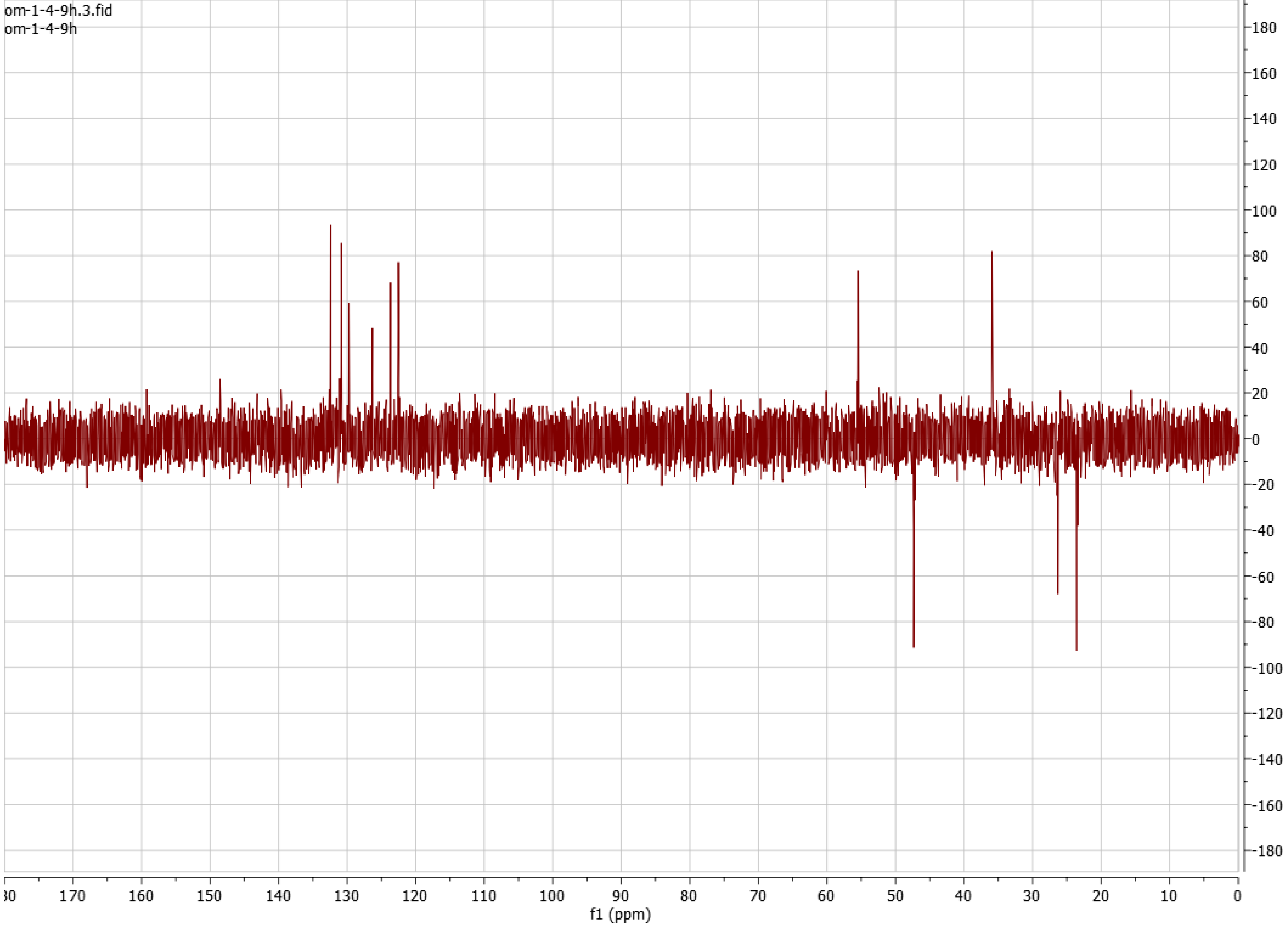


**SI 4:** DEPT-135 spectrum of 4b [100 MHz, DMSO-*d6* ]

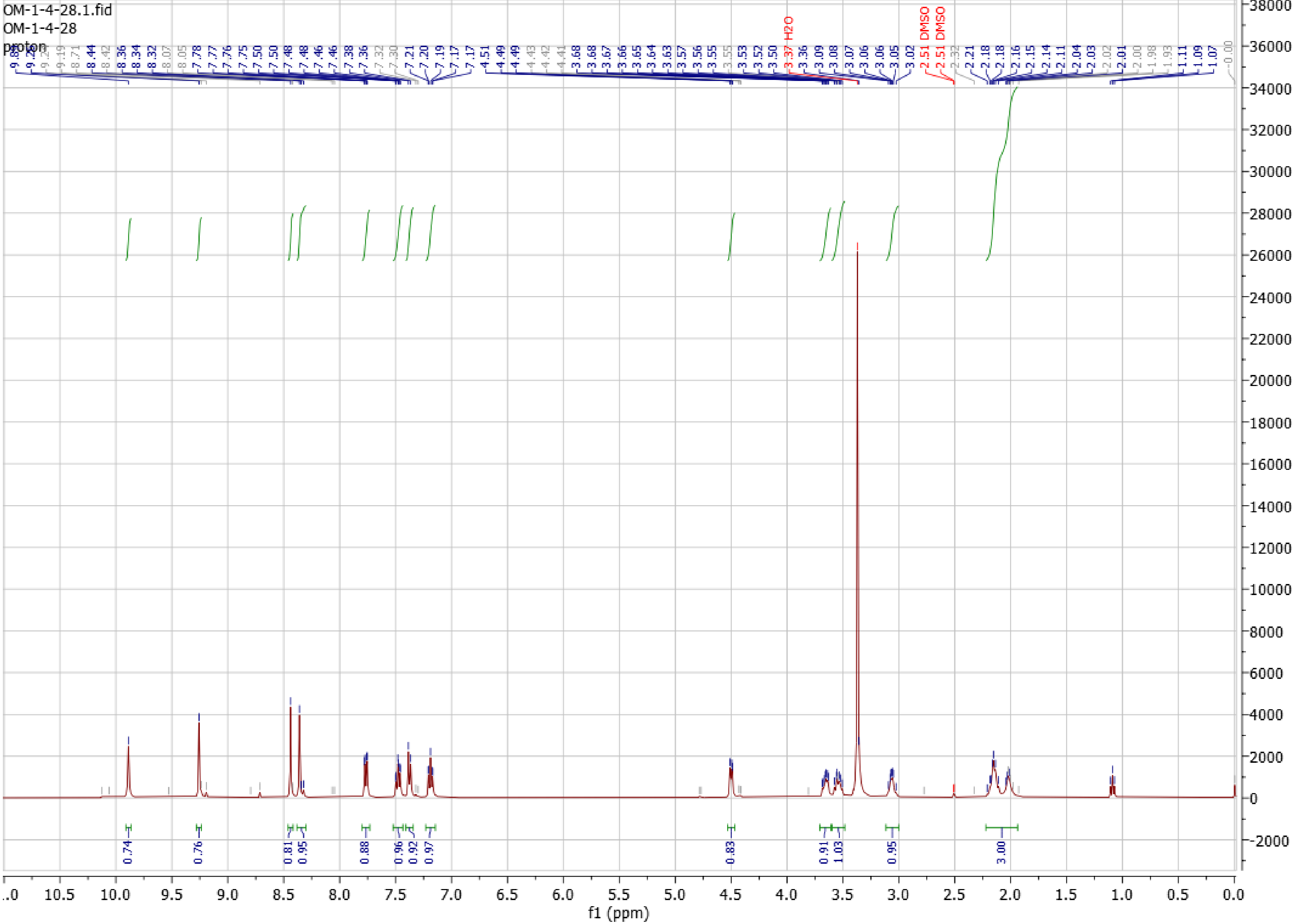


**SI 5:** $^1$H NMR spectrum of 4c [400 MHz, DMSO-*d6* ]

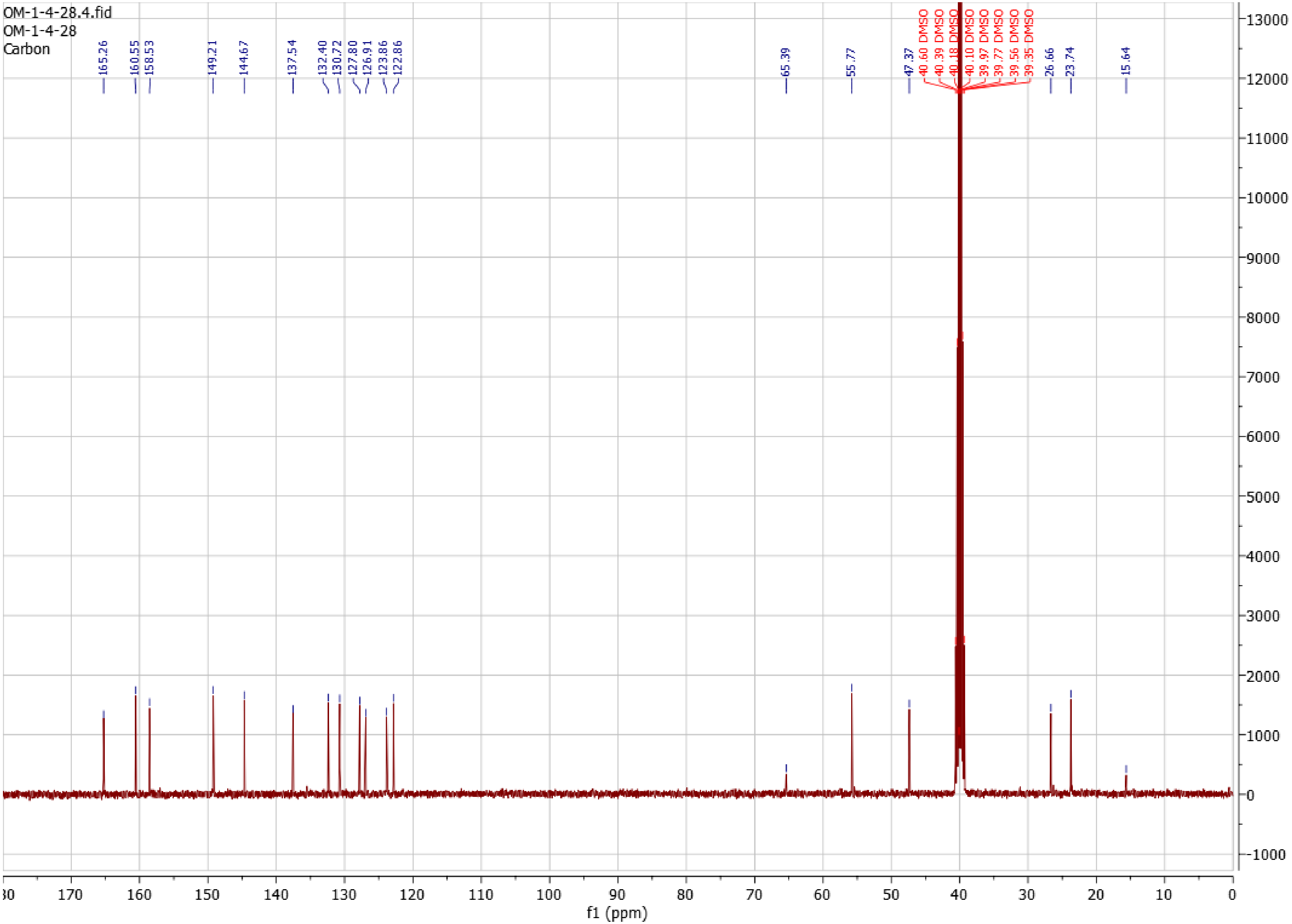


**SI 6:** $^{13}$C NMR spectrum of 4c [400 MHz, DMSO-*d6* ]

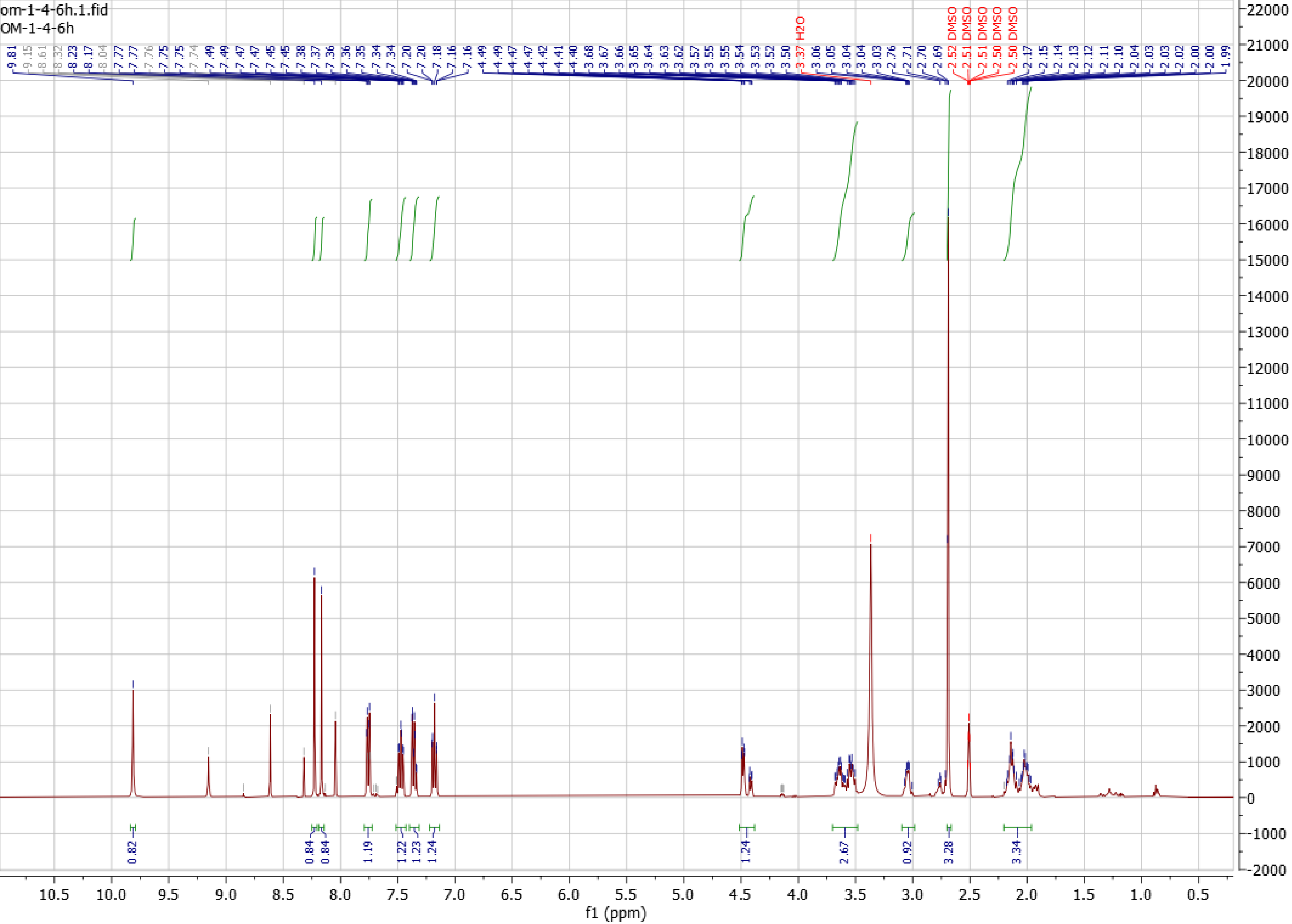

**SI 7:** $^{1}$H NMR spectrum of 4d [400 MHz, DMSO-*d6*]

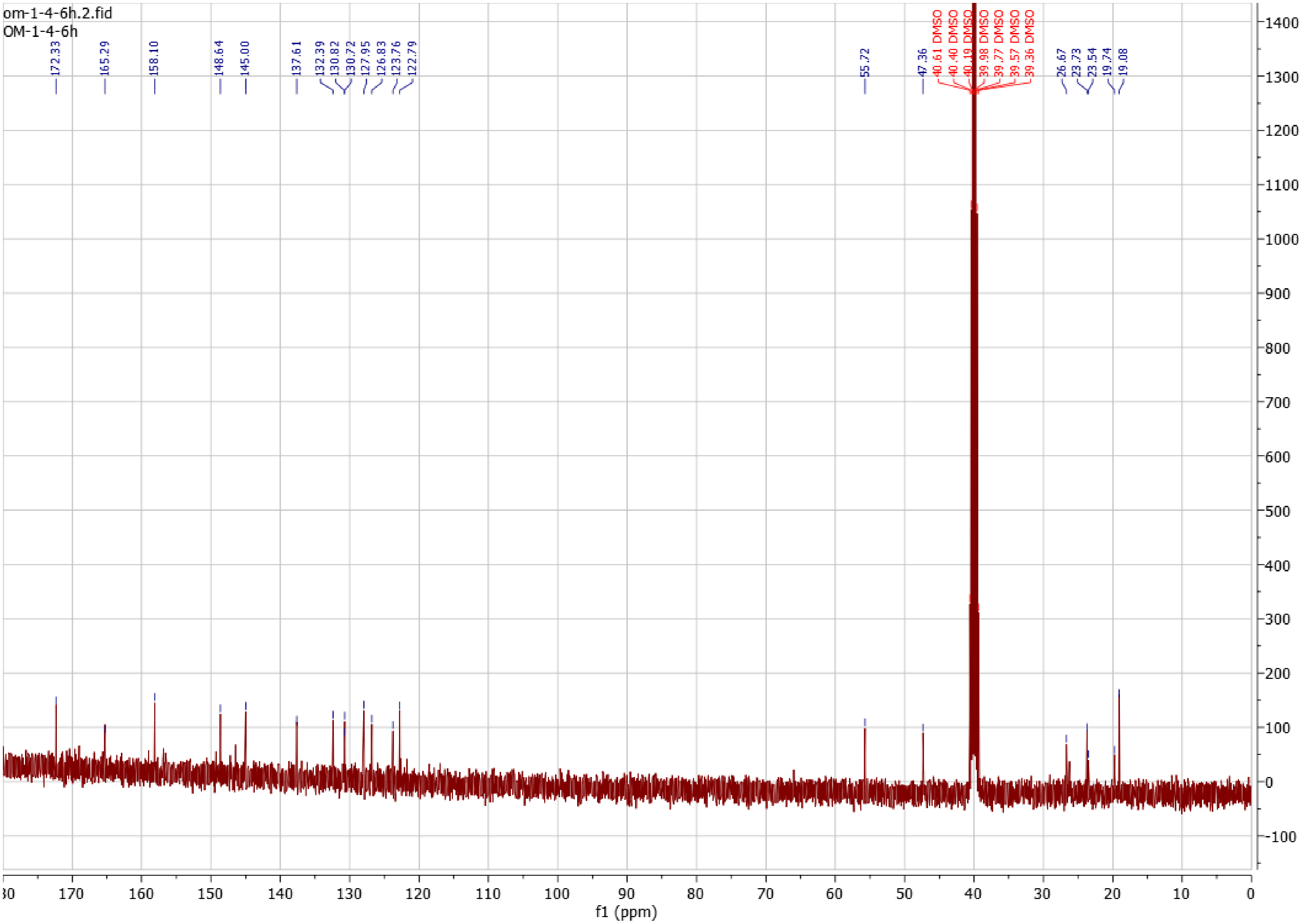


**SI 8:** $^{13}$C NMR spectrum of 4 d [400 MHz, DMSO-*d6*]

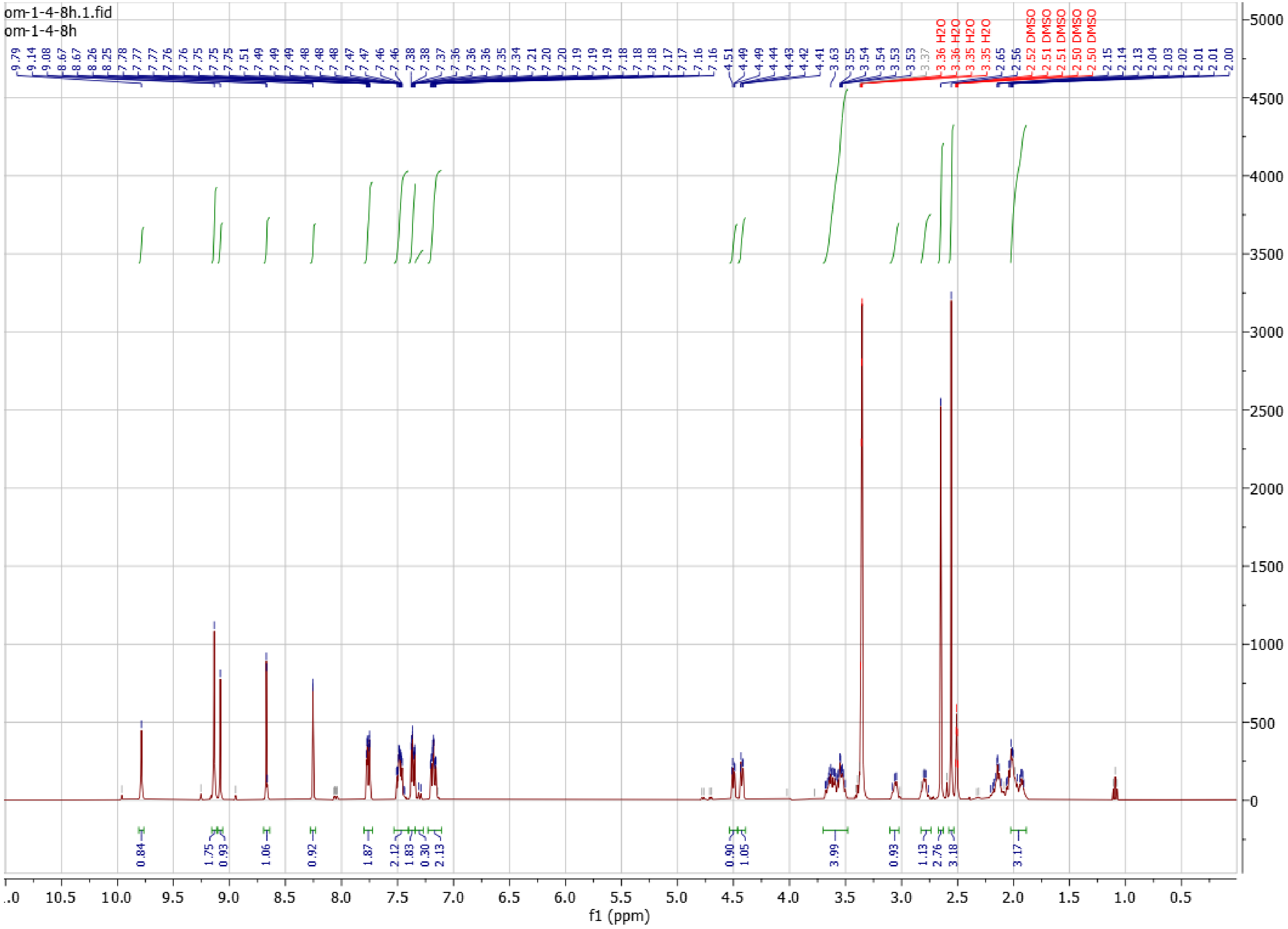

**SI 9:** $^{1}$H NMR spectrum of 4e [400 MHz, DMSO-*d6*]

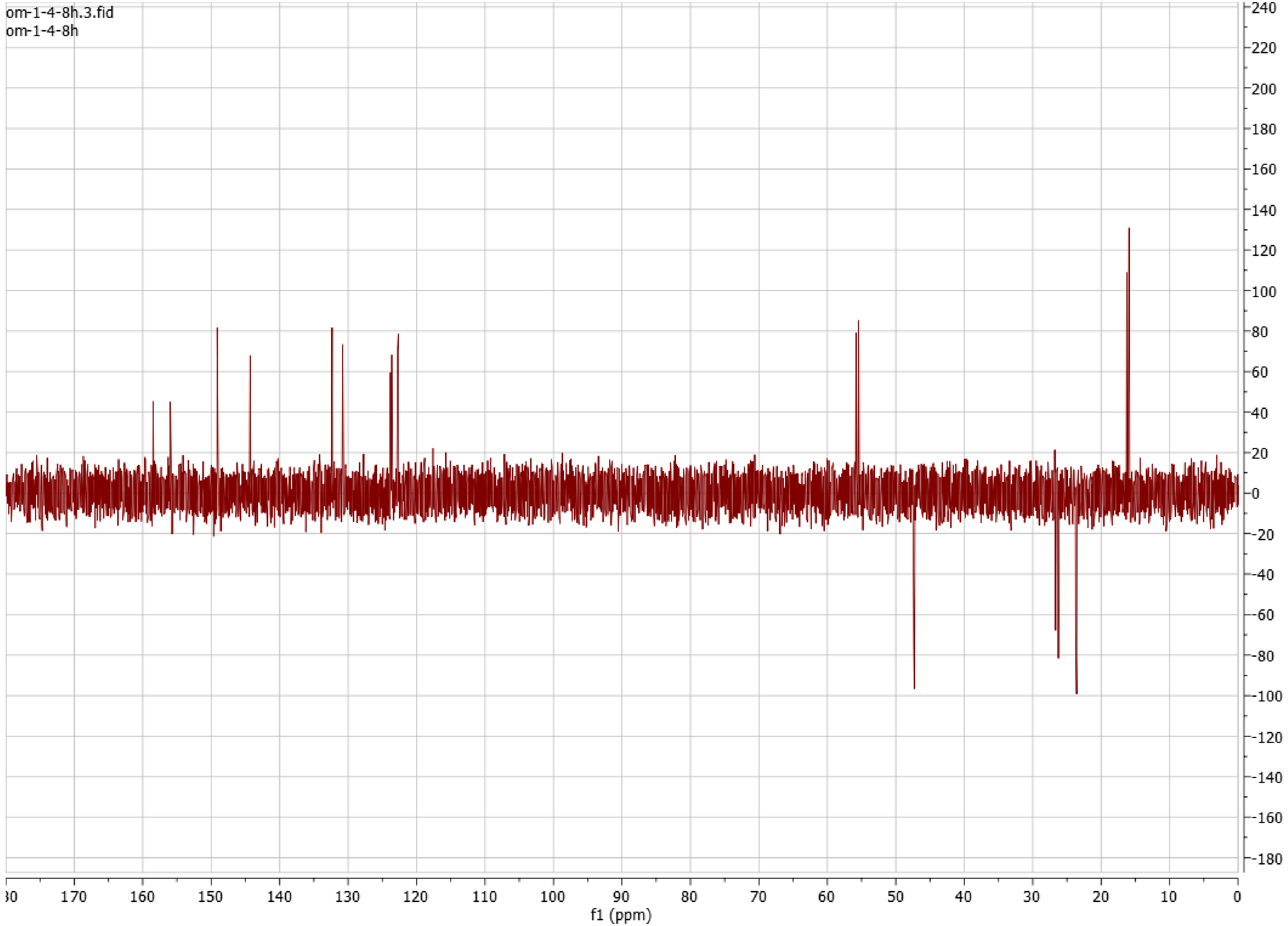


**SI 10:** Dept-135 NMR spectrum of 4 e  [100 MHz, DMSO-*d6*]

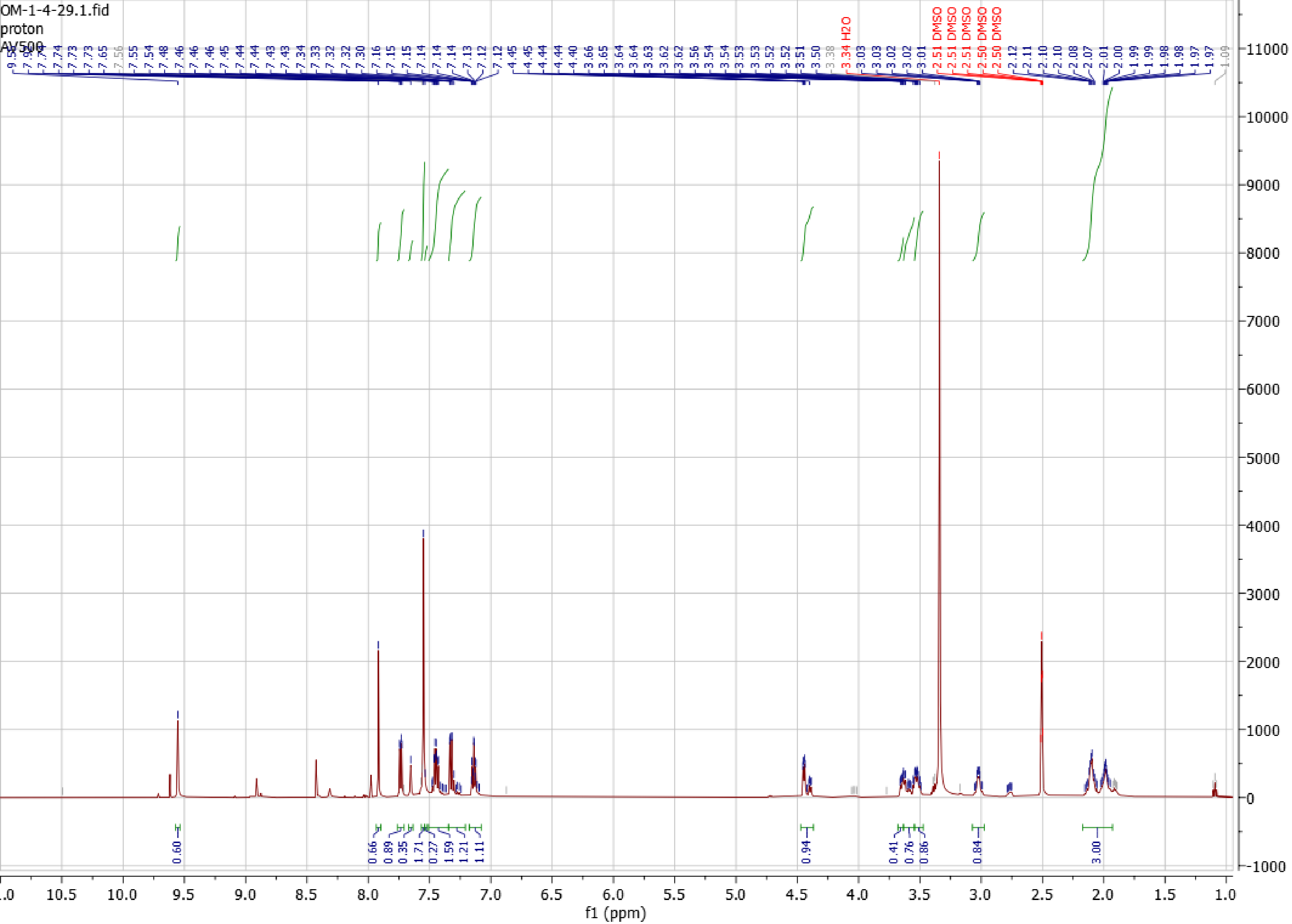


**SI 11:** 1H NMR spectrum of 4f [500 MHz, DMSO-*d6*]

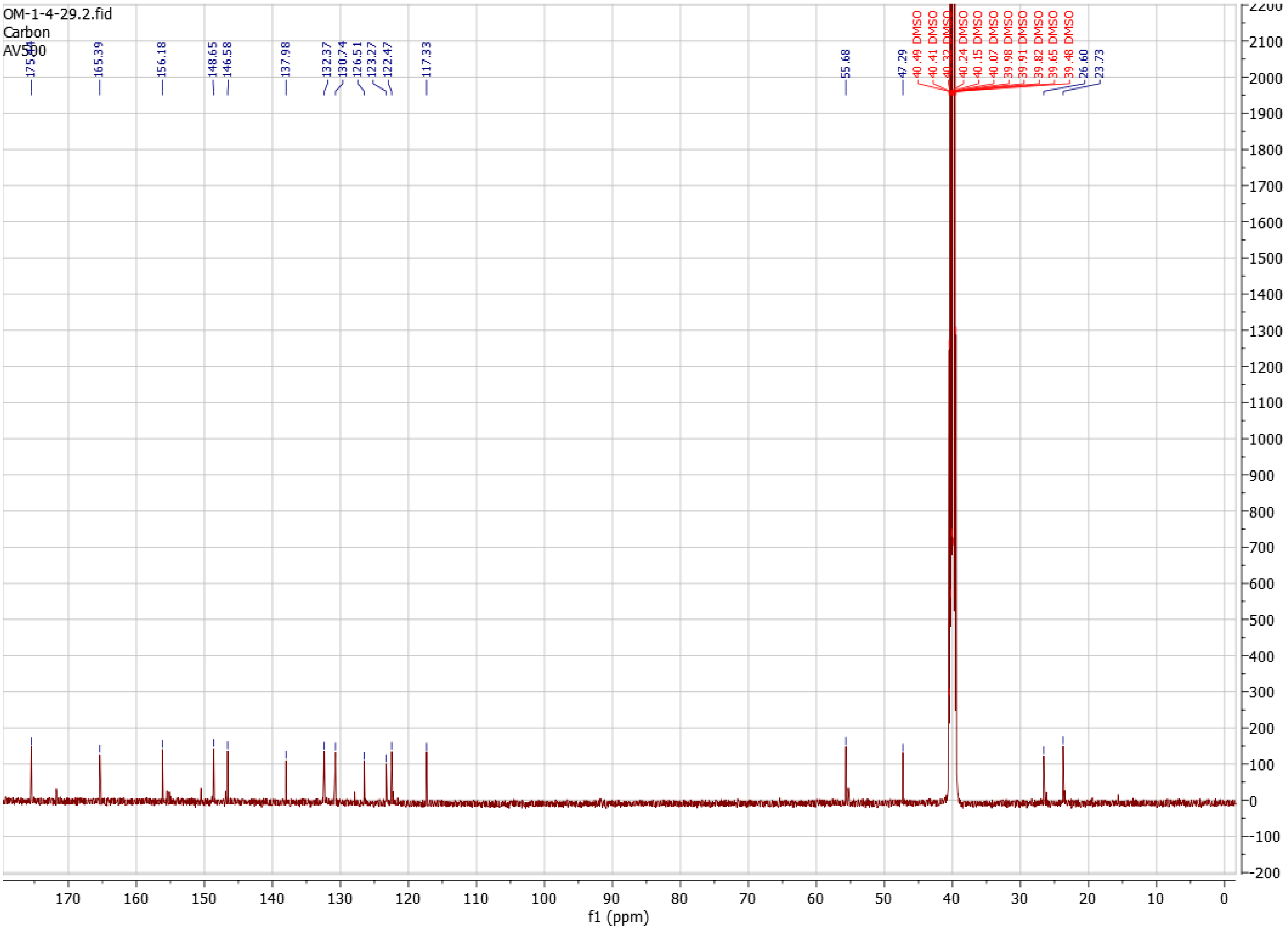


**SI 12:** $^{13}$C NMR spectrum of 4f [125 MHz, DMSO-*d6*]

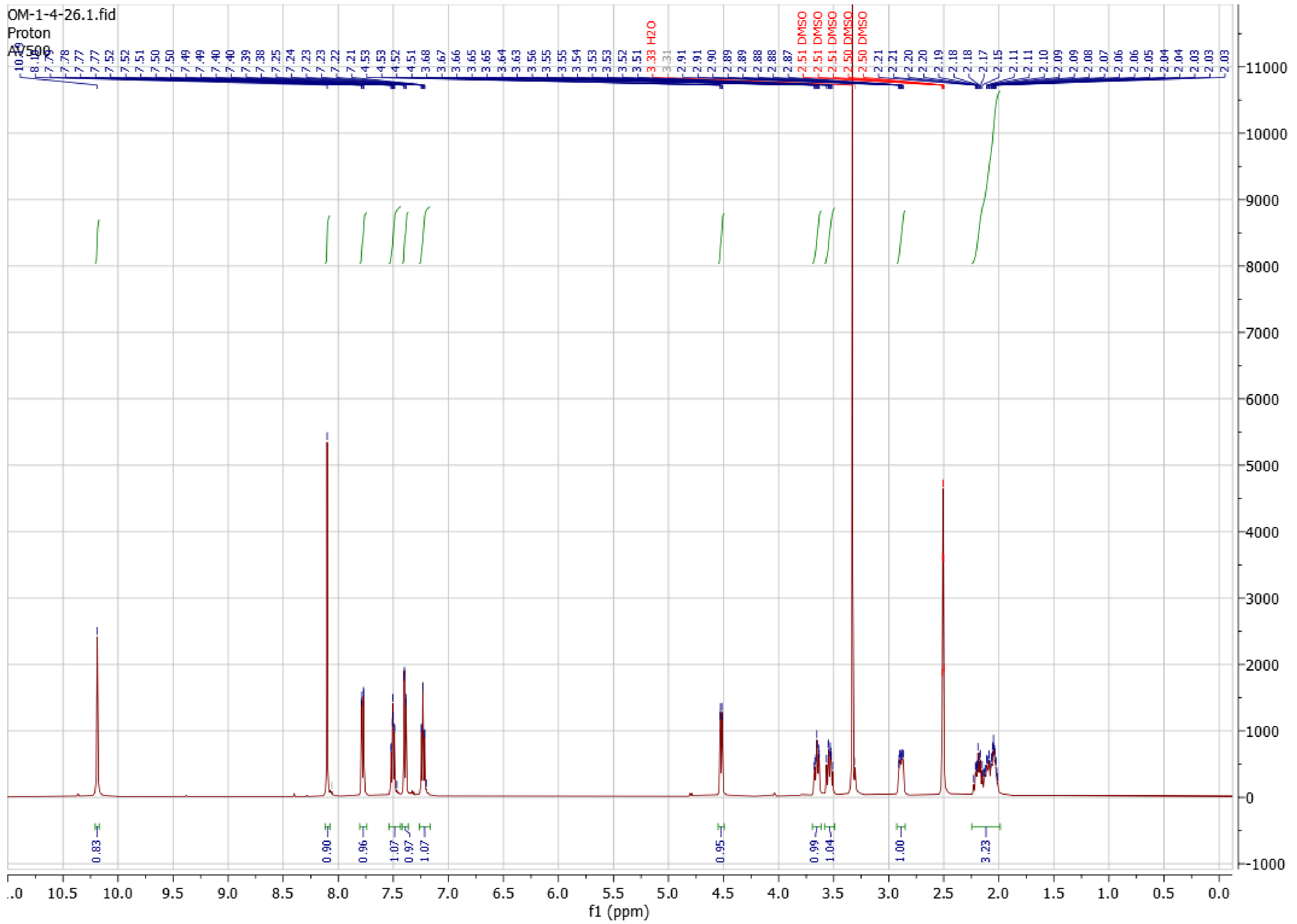

**SI 13:** $^{1}$H NMR spectrum of 4g [500 MHz,  DMSO-*d6*]

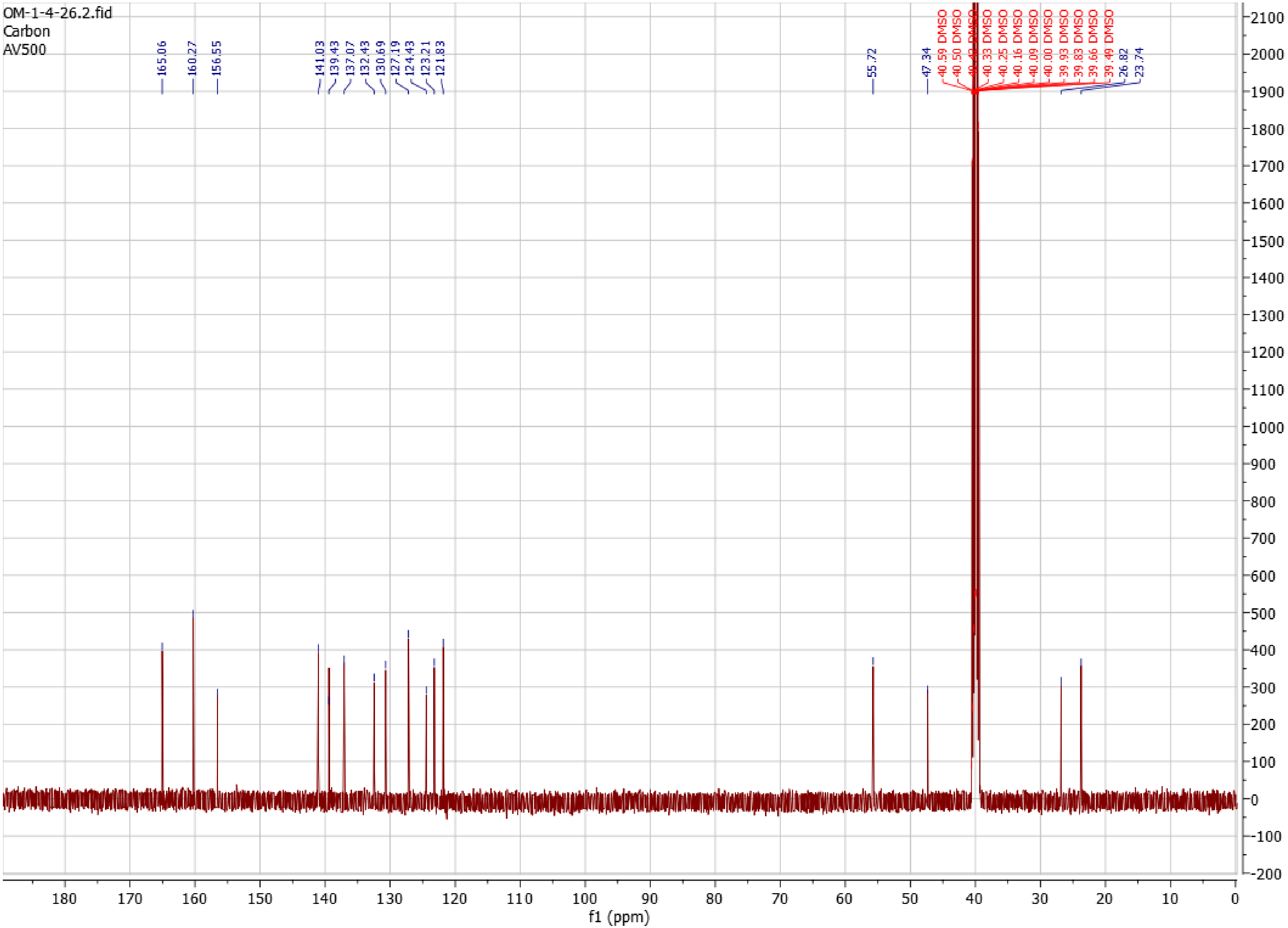


**SI 14:** $^{13}$C NMR spectrum of 4g [125 MHz, DMSO-*d6*]

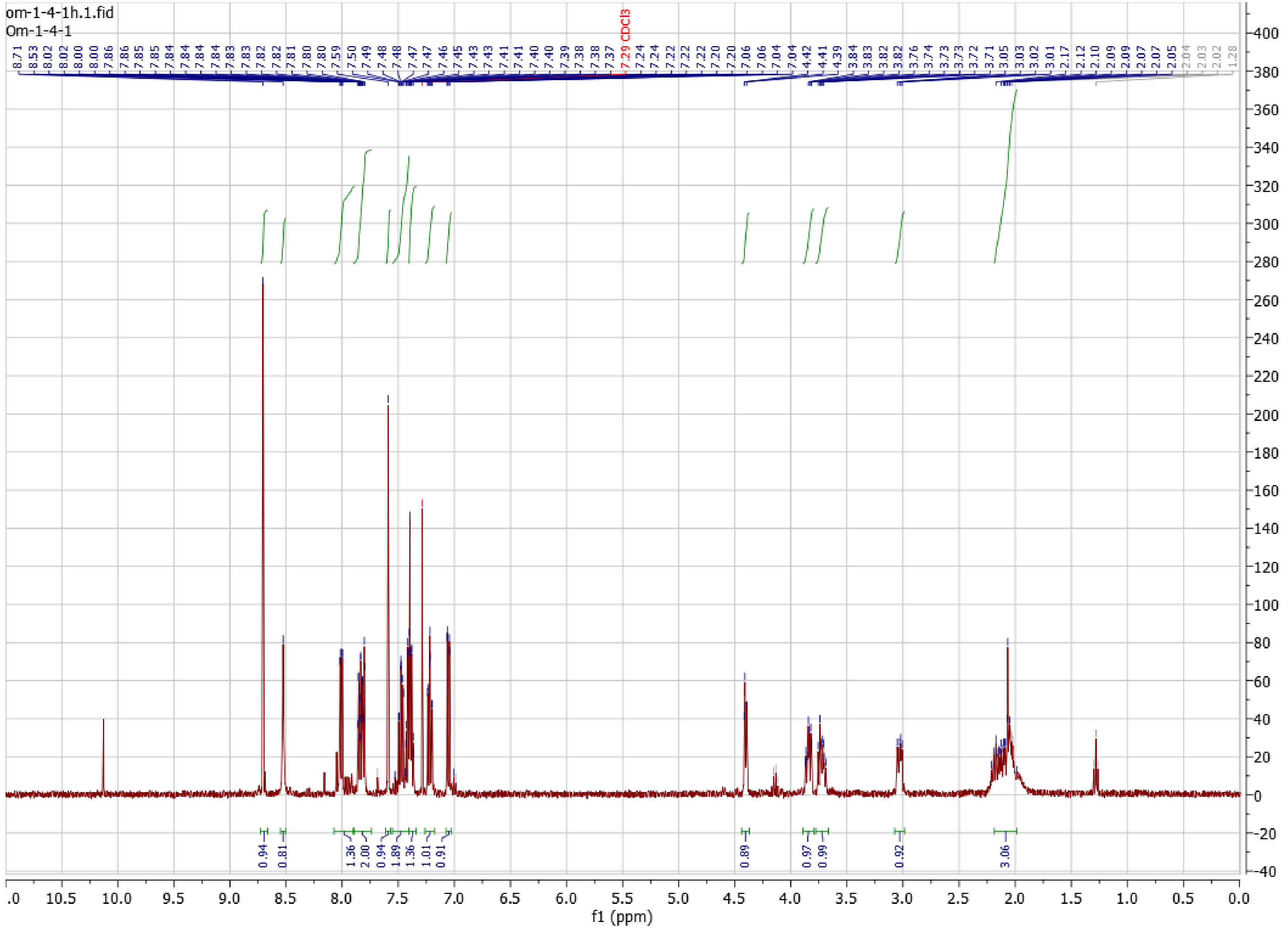

**SI 15:** $^{1}$H NMR spectrum of 4h [400 MHz, $CDCl_3$-*d*]

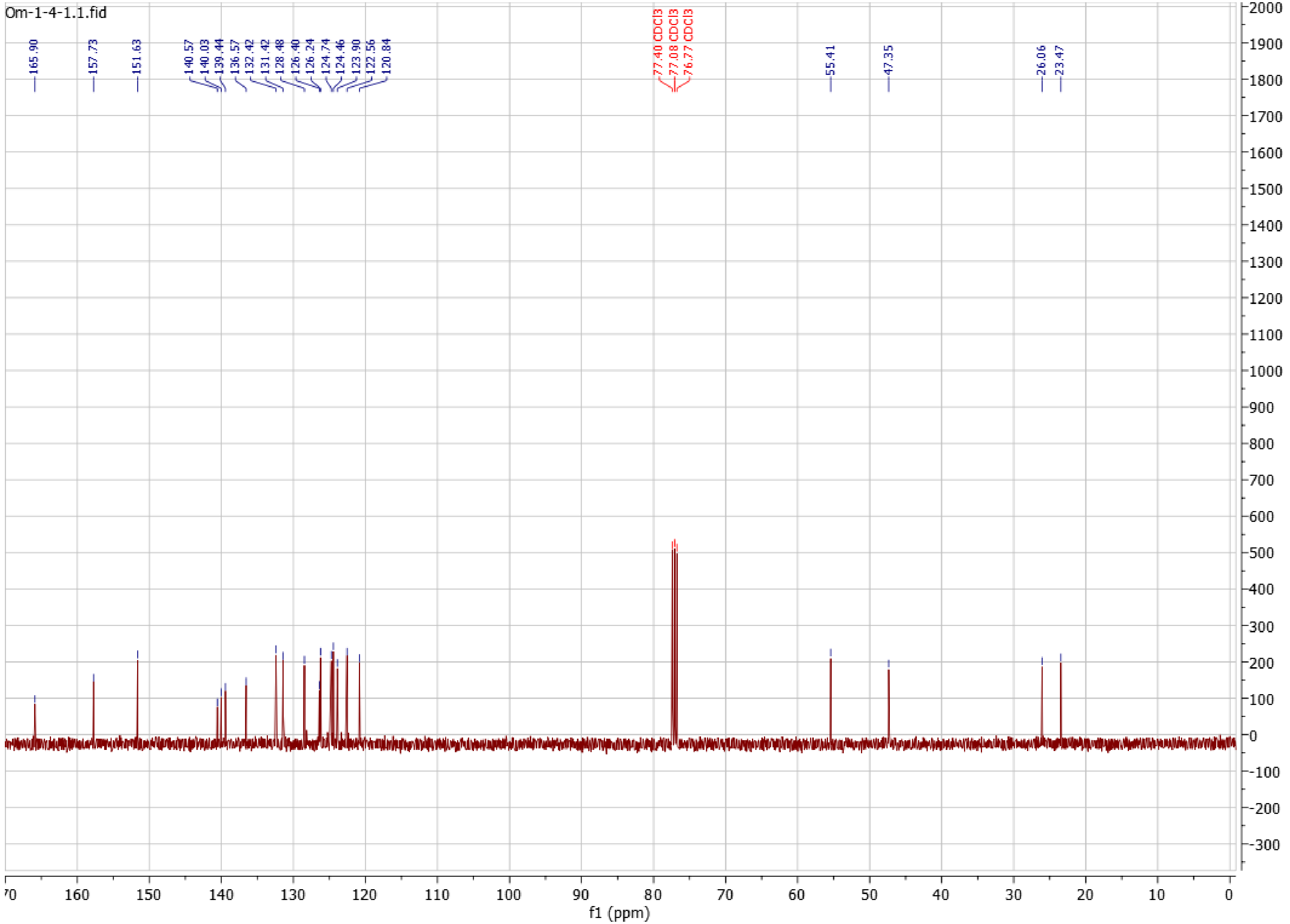


**SI 16:** $^{13}$C NMR spectrum of 4h [100 MHz, $CDCl_3$-*d*]

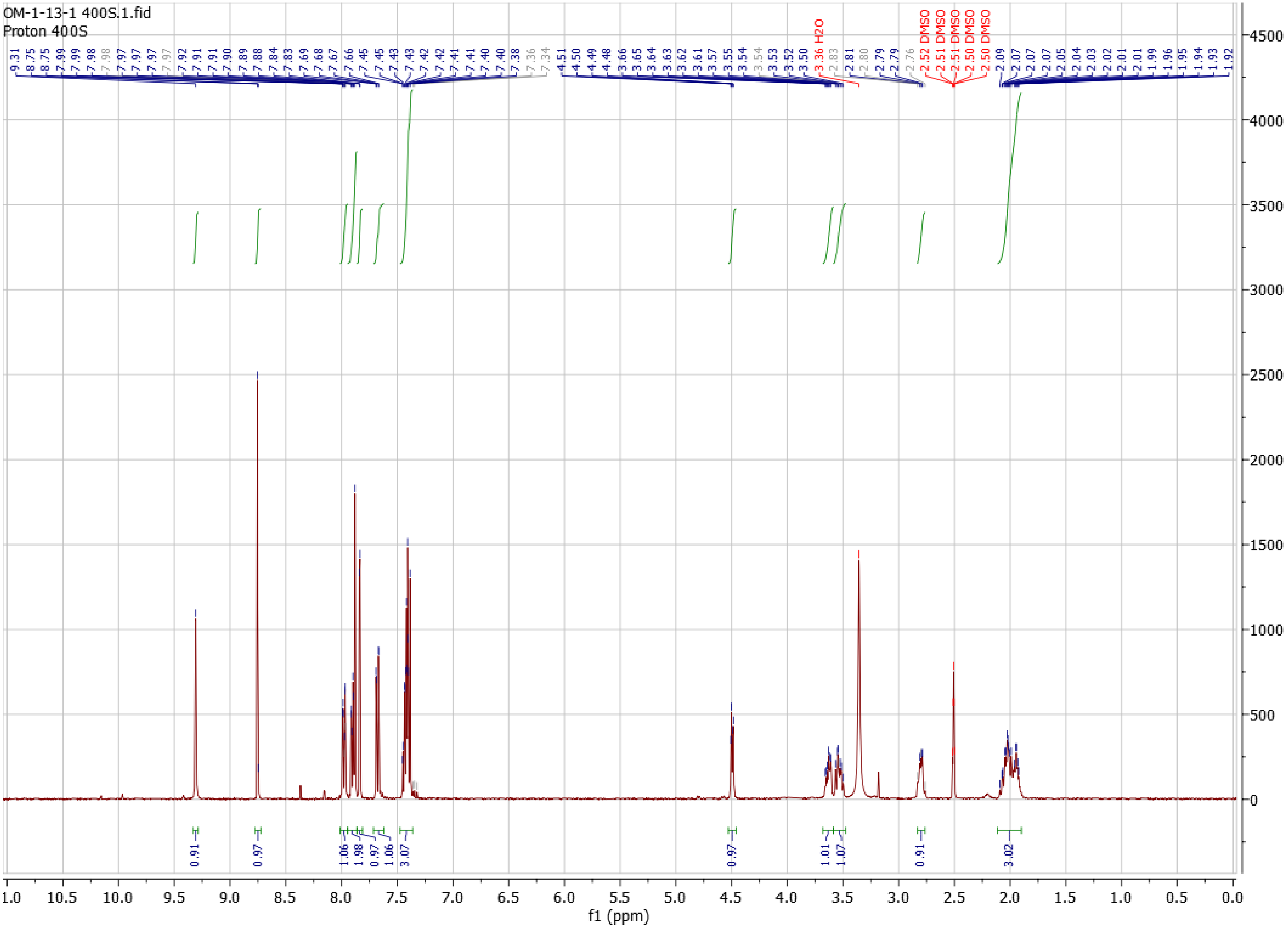


**SI 17:** $^{1}$H NMR spectrum of 4i [400 MHz, DMSO-*d6*]

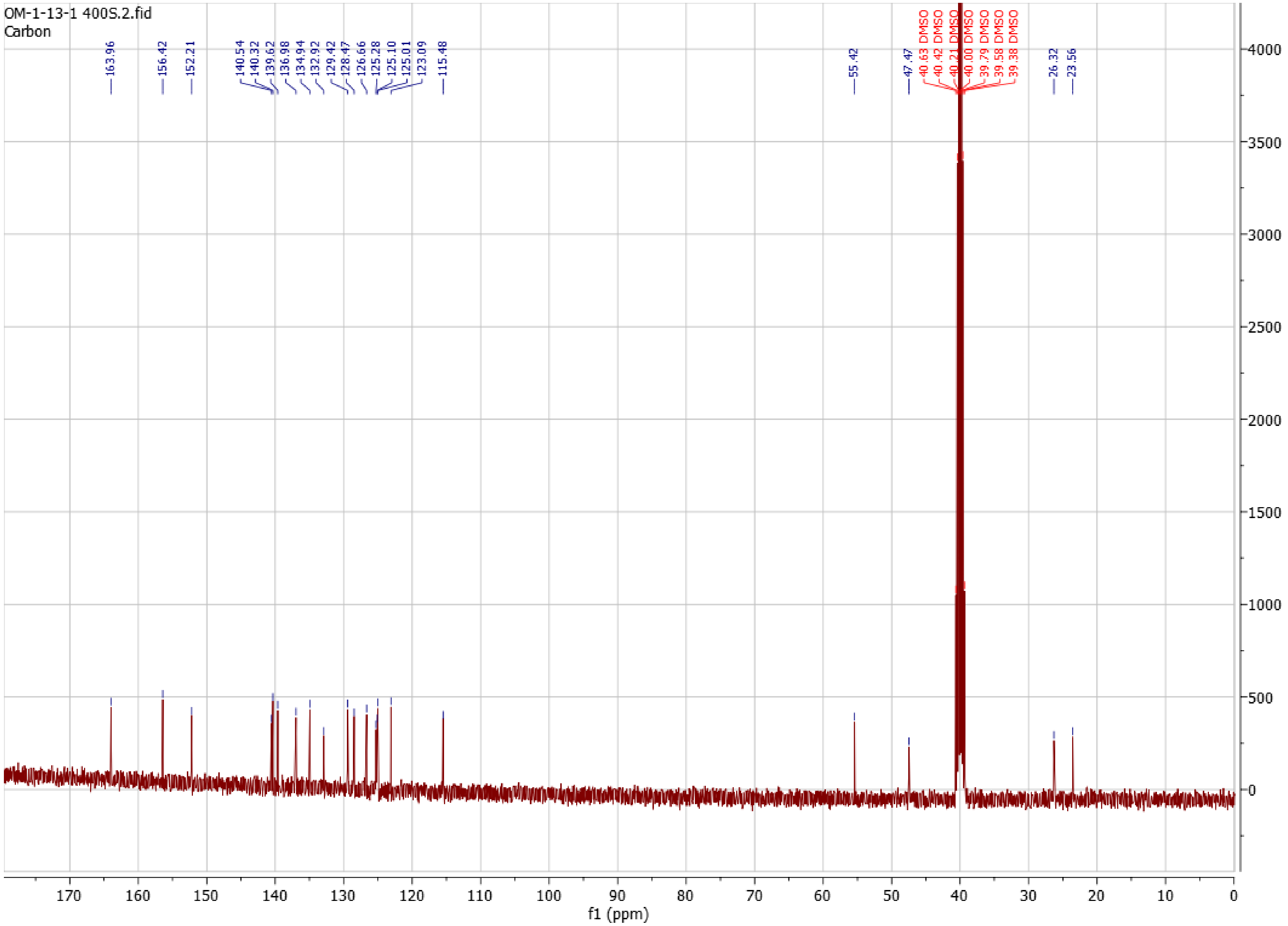


**SI 18:** $^{13}$C NMR spectrum of 4i [400 MHz, DMSO-*d6*]

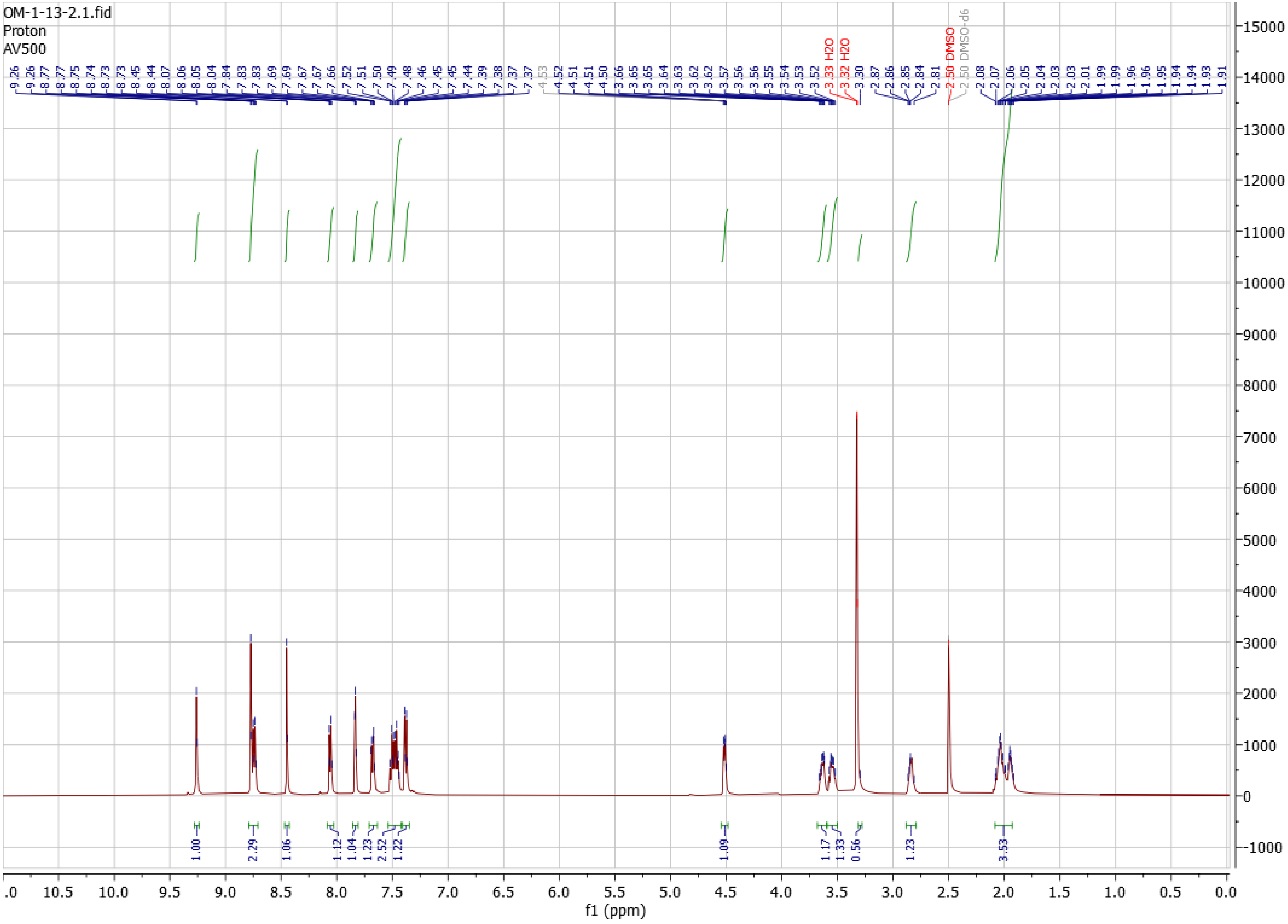

**SI 19:** $^{1}$H NMR spectrum of 4j [500 MHz, DMSO-*d6*]

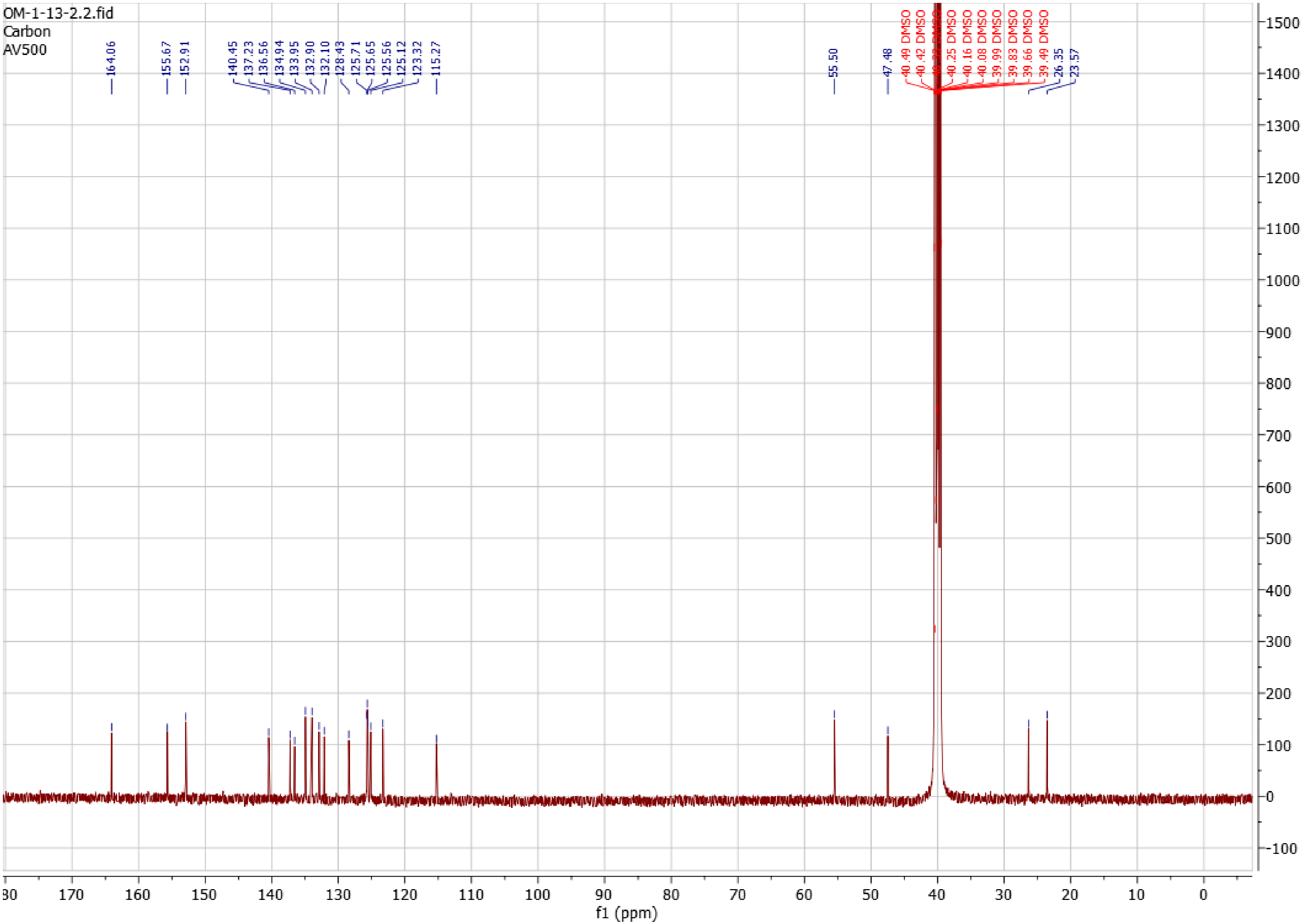


**SI20:** $^{13}$C NMR spectrum of 4j [125 MHz, DMSO-*d6*

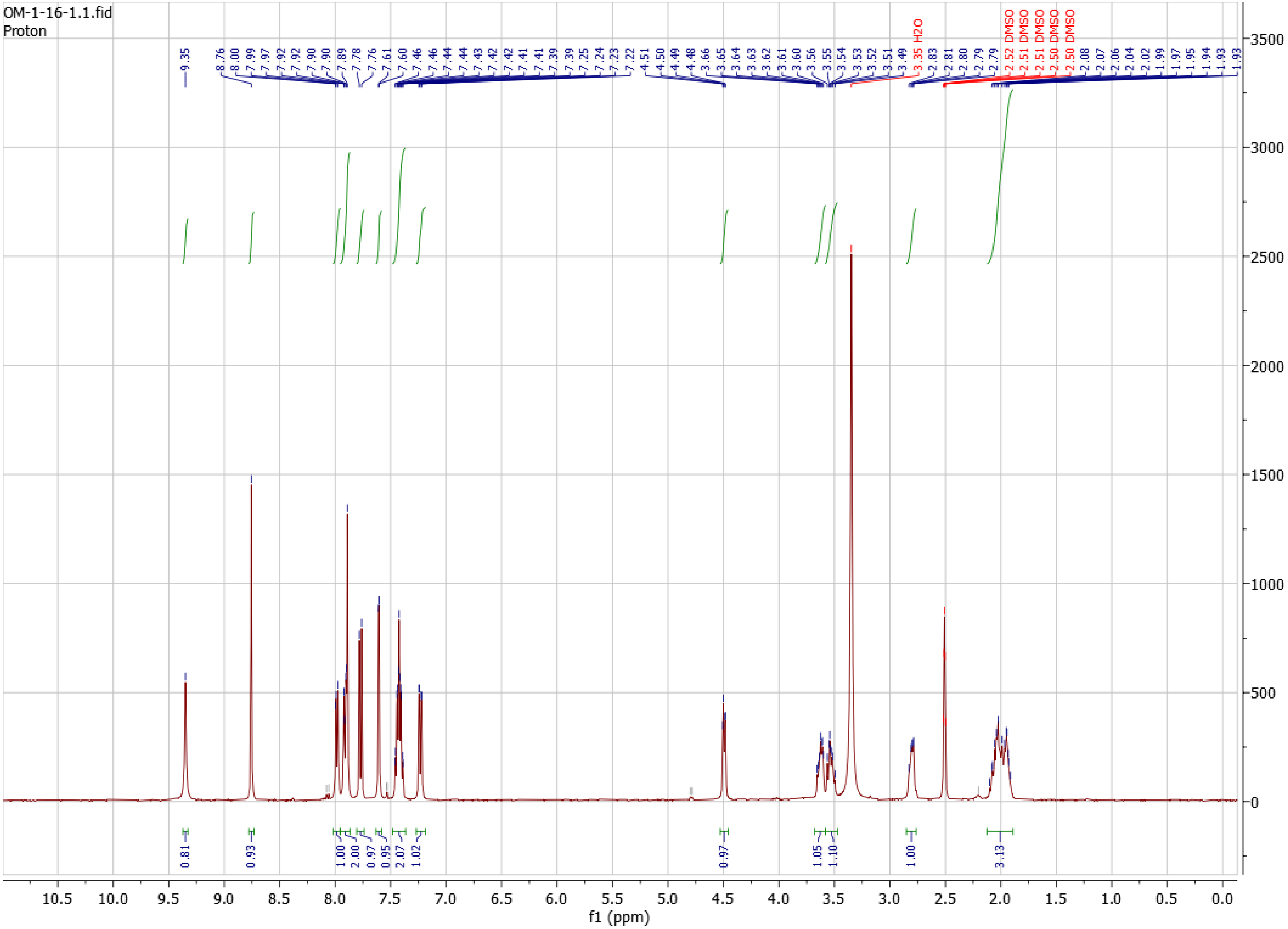

**SI21:** $^{1}$H NMR spectrum of 4k [400 MHz, DMSO-*d6*]

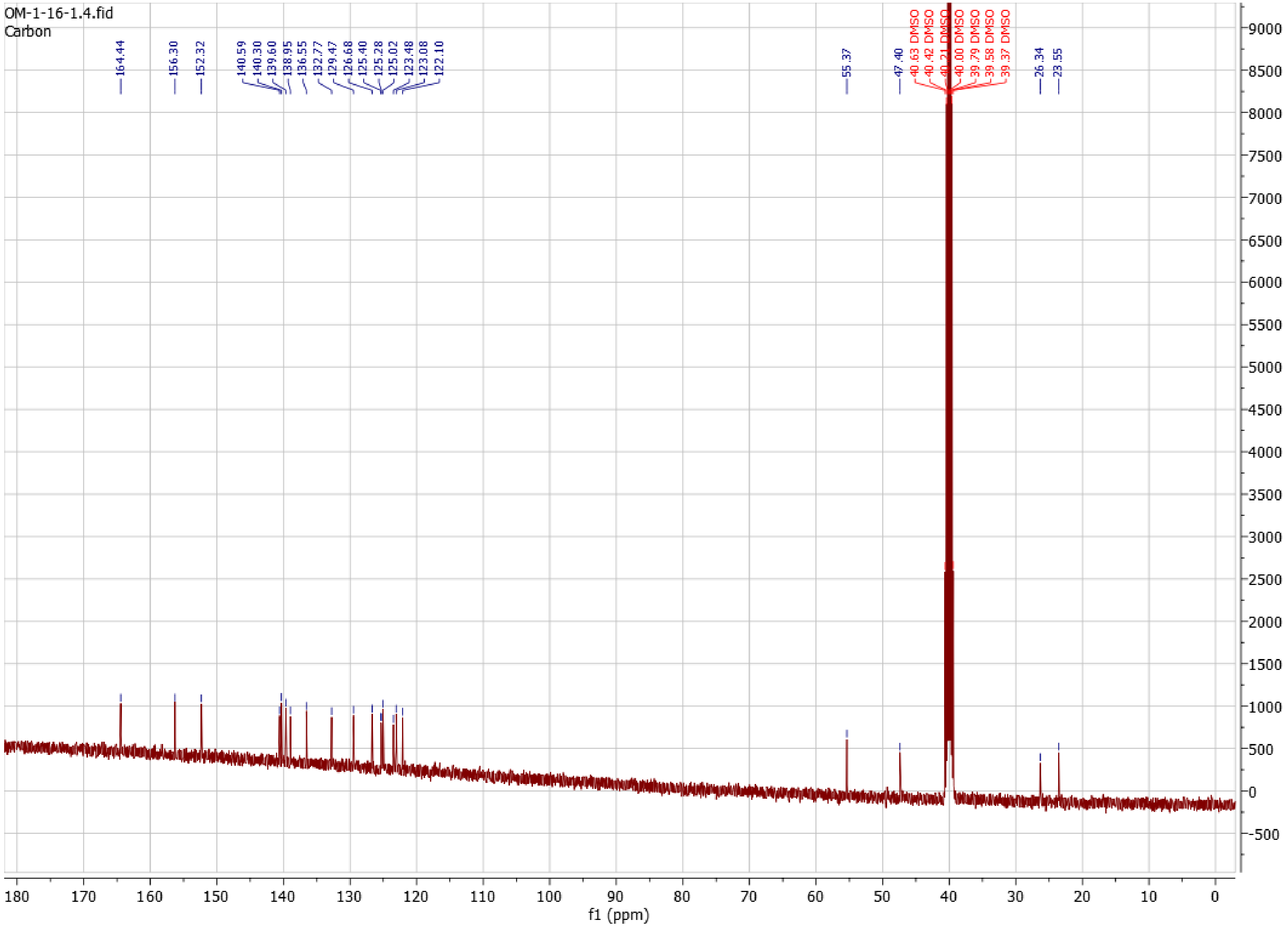


**SI22:** $^{13}$C NMR spectrum of 4k [100 MHz, DMSO-*d6*]

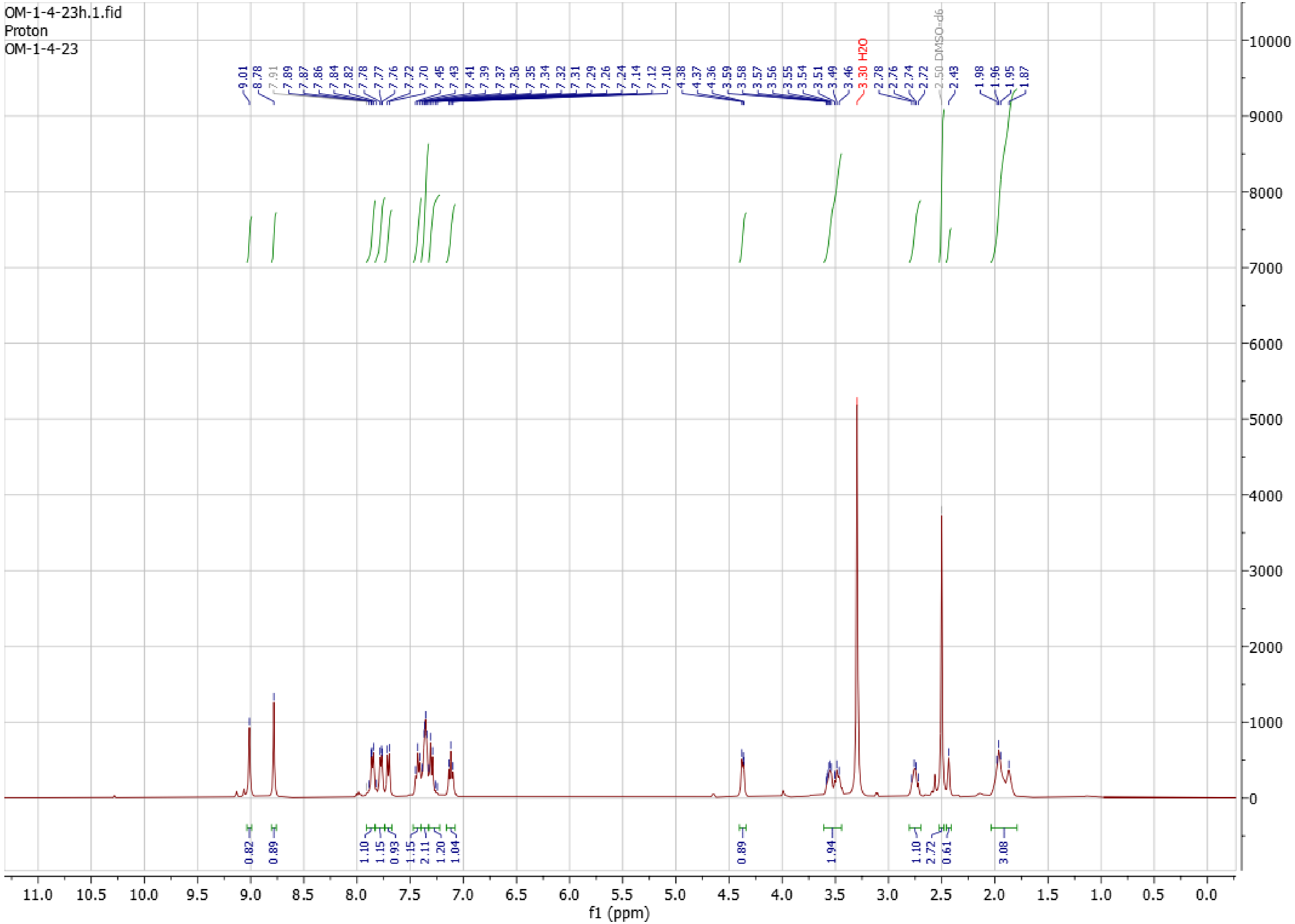


**SI23:** $^{1}$H NMR spectrum of 4l [400 MHz, DMSO-*d6*]

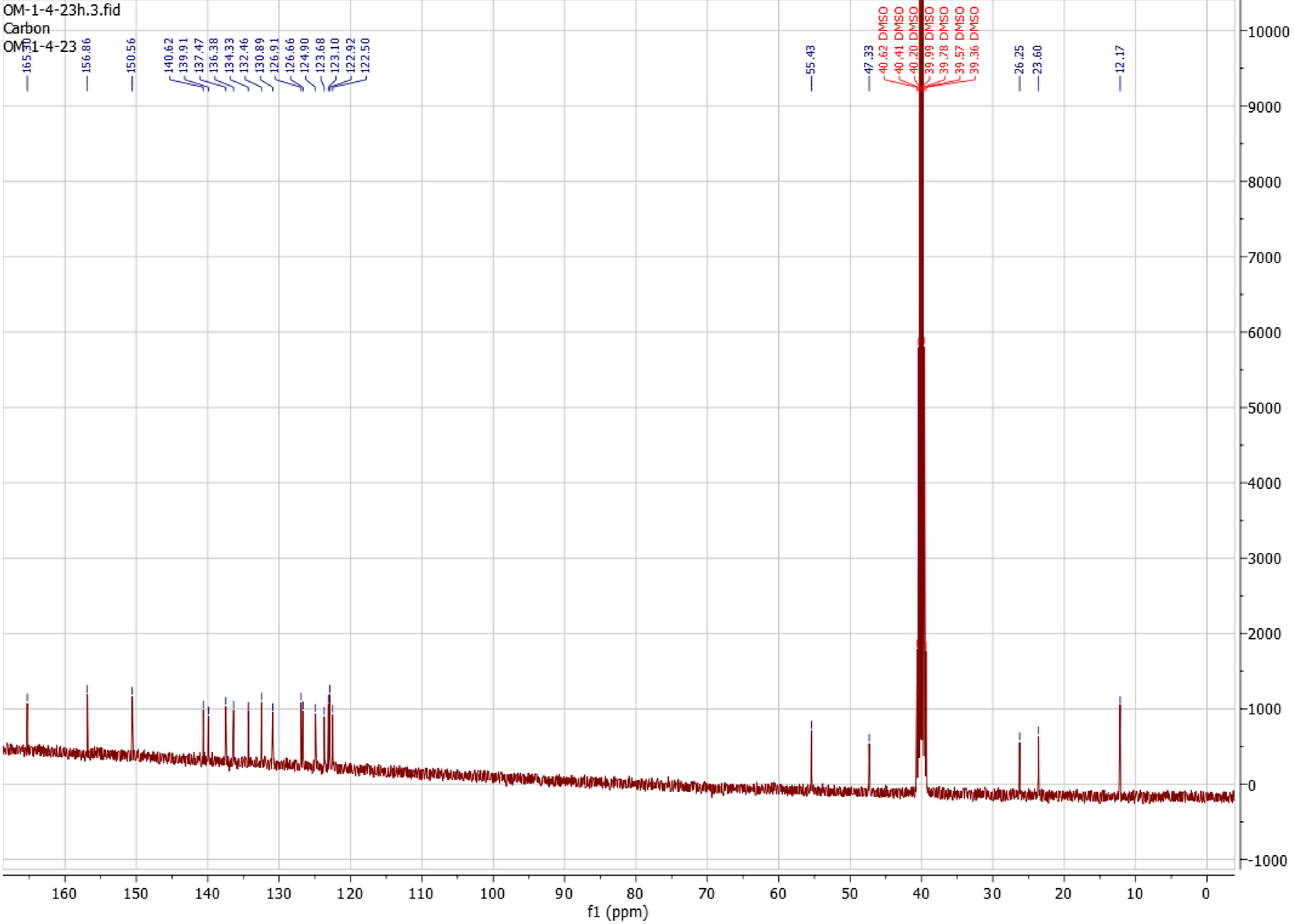


**SI24:** $^{13}$C NMR spectrum of 4l [100 MHz, DMSO-*d6*]

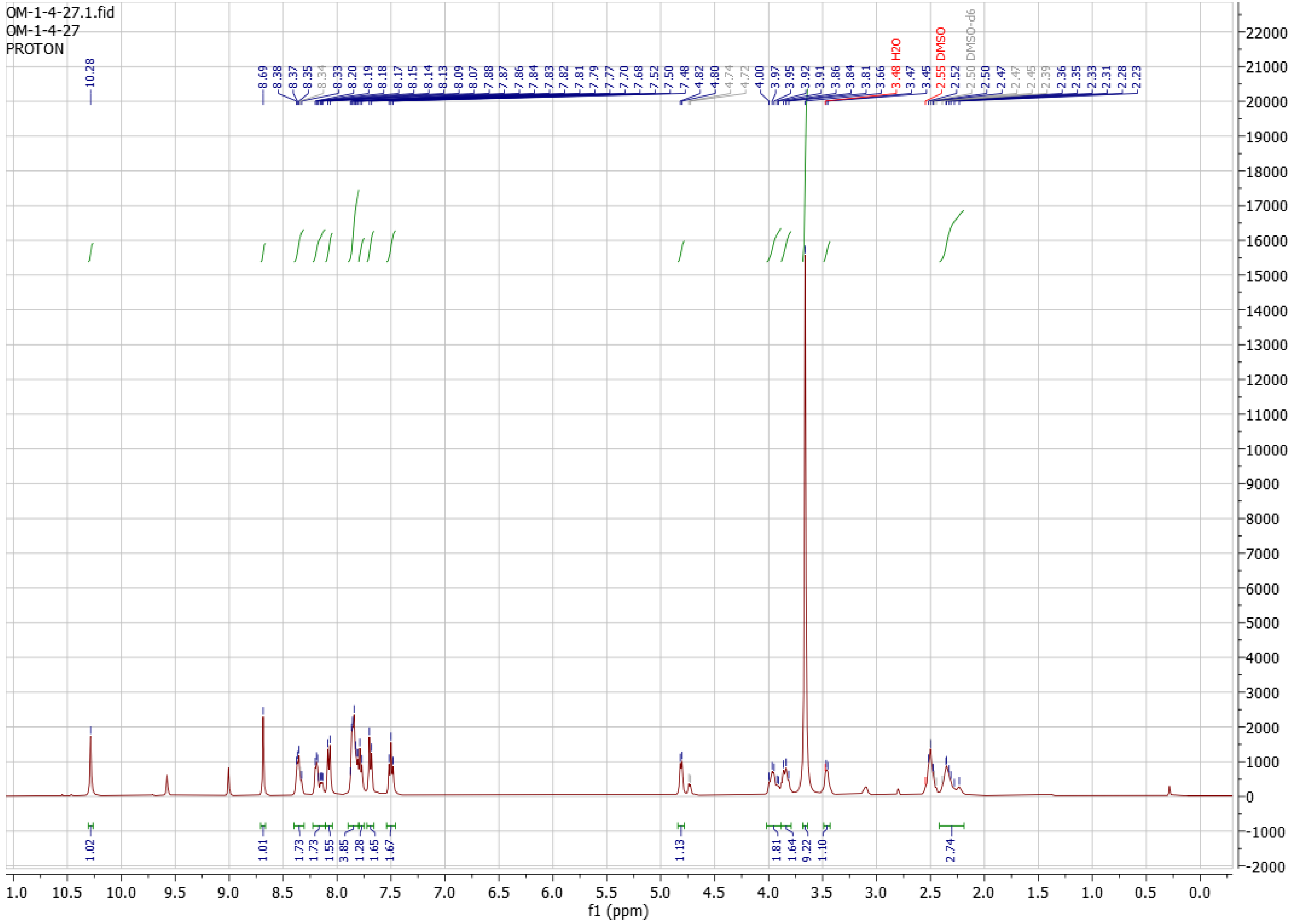


**SI25:** $^{1}$H NMR spectrum of 4m [400 MHz, DMSO-*d6*]

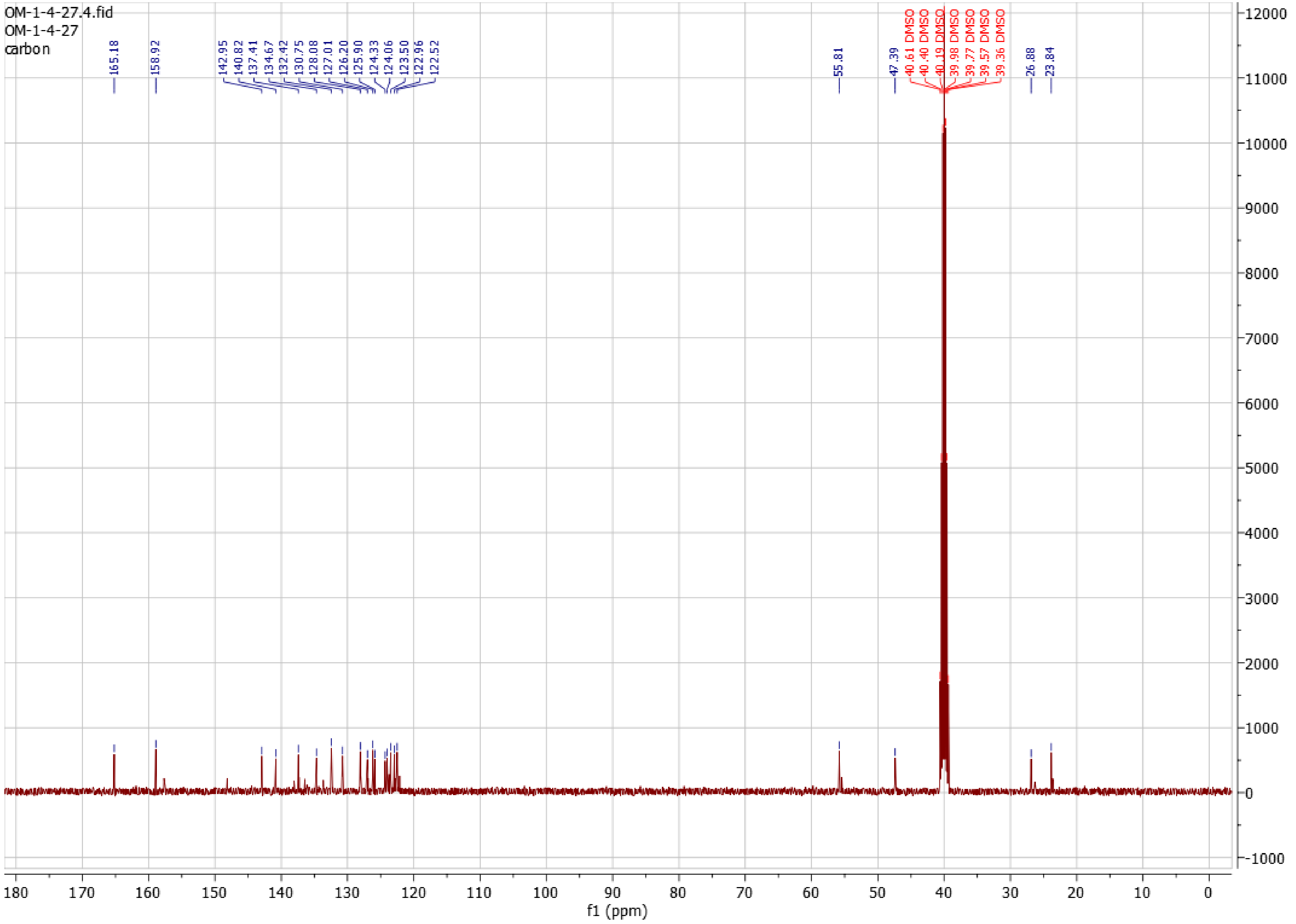


**SI26:** $^{13}$C NMR spectrum of 4m [100 MHz, DMSO-*d6*]

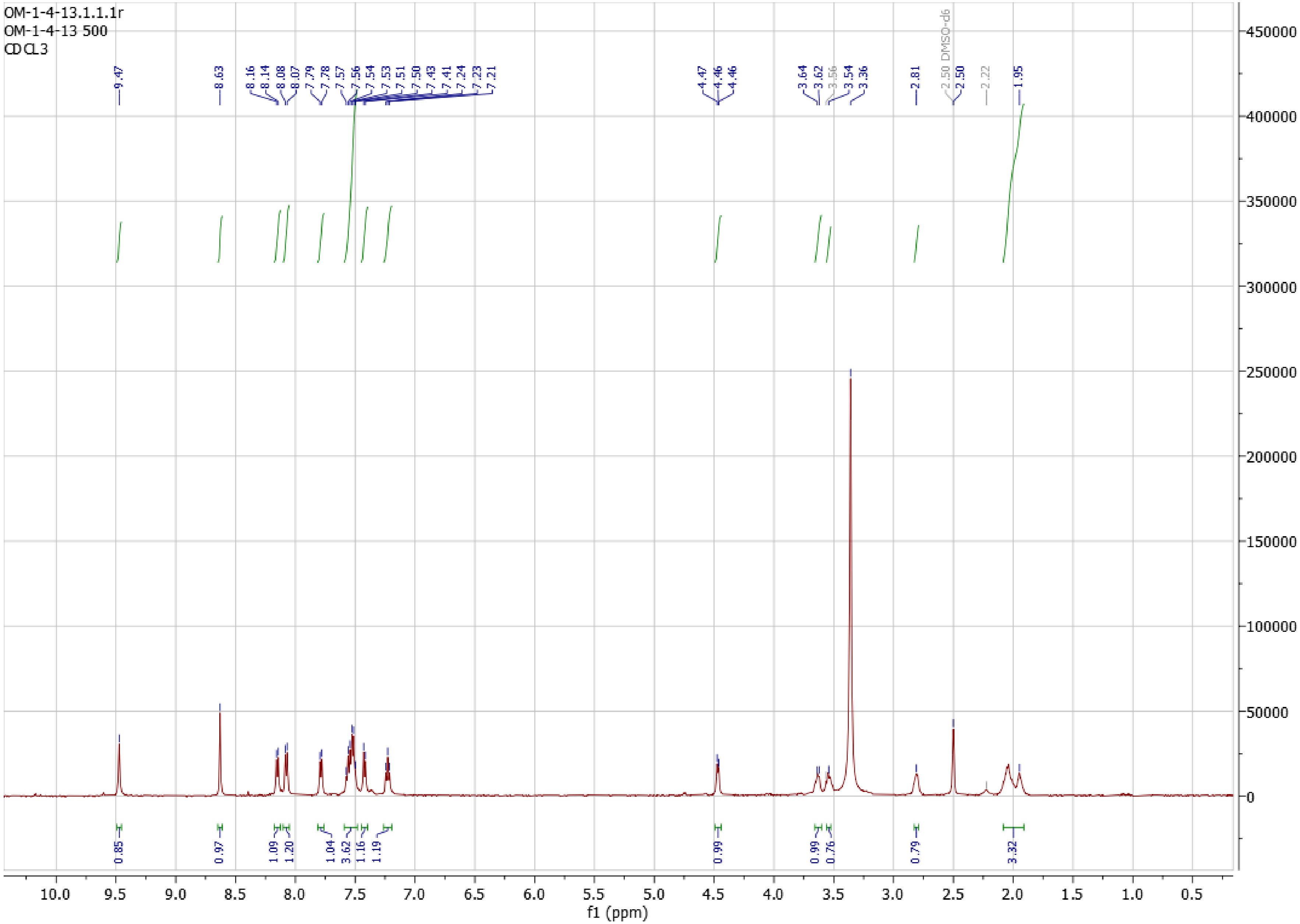


**SI27:** $^{1}$H NMR spectrum of 4n [500 MHz, DMSO-*d6*]

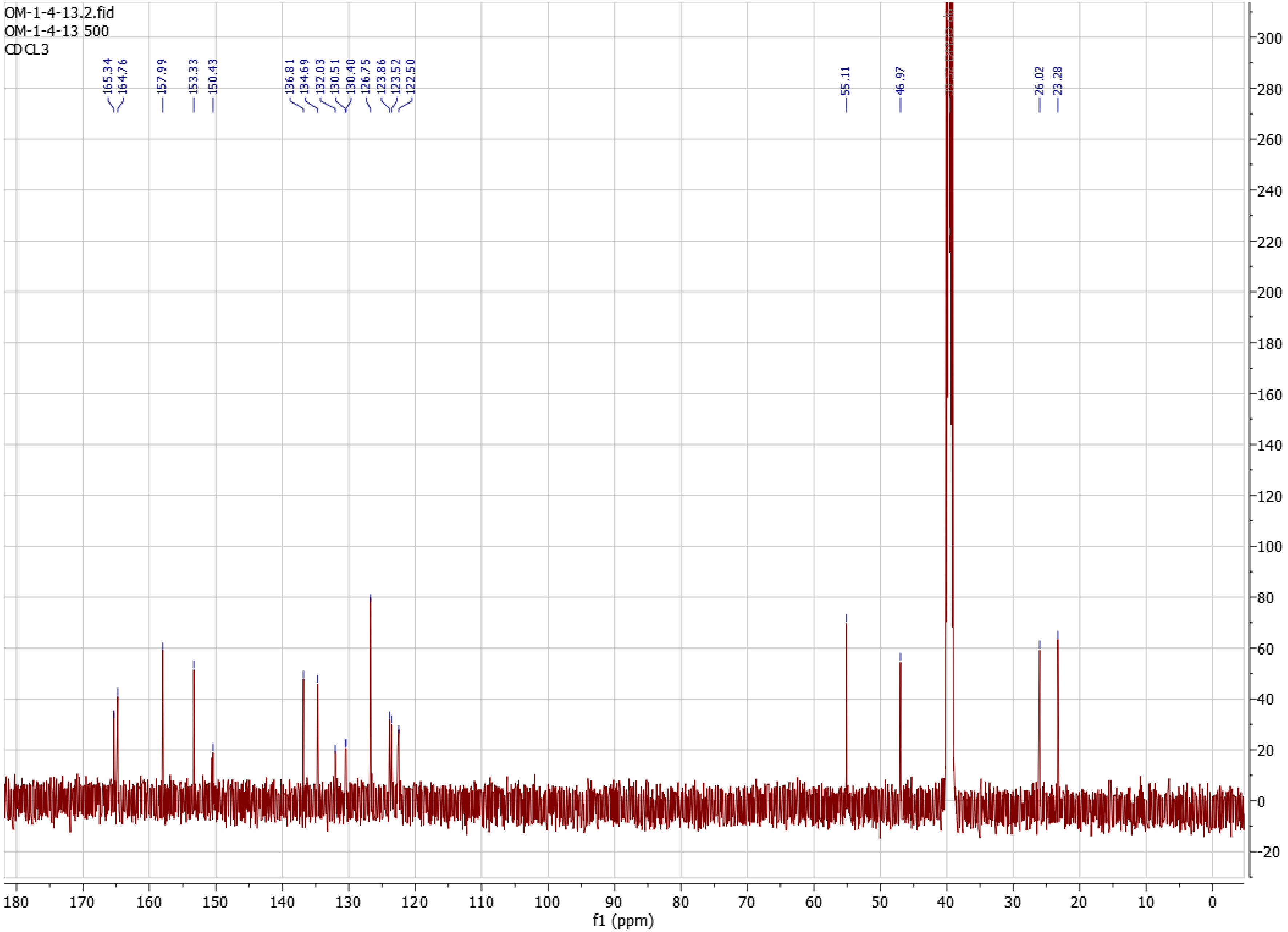


**SI28:** $^{13}$C NMR spectrum of 4n [125 MHz, DMSO-*d6*]

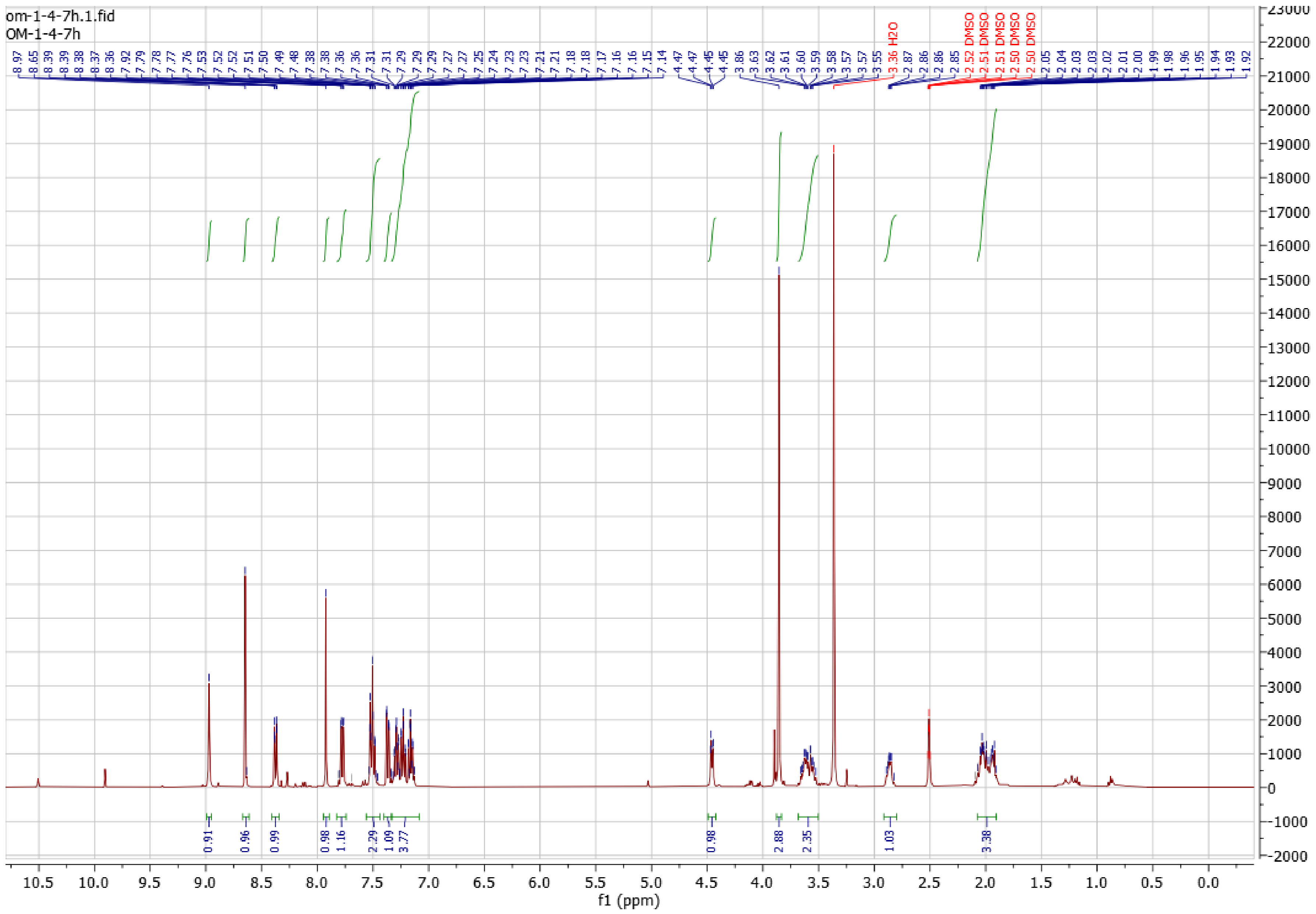

**SI29:** $^{1}$H NMR spectrum of 4o [400 MHz, DMSO-*d6*]

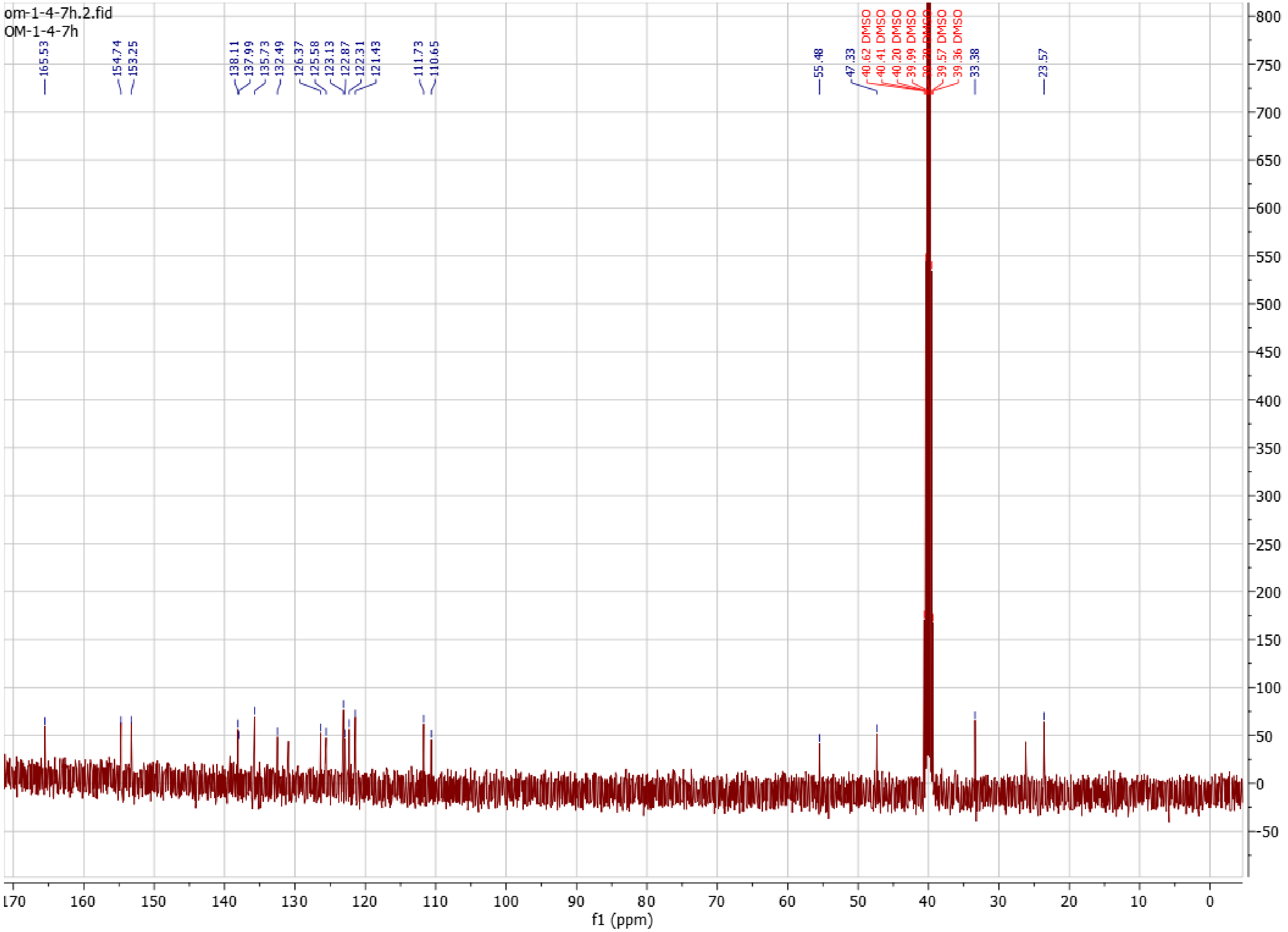


**SI30:** $^{13}$C NMR spectrum of 4o [100 MHz, DMSO-*d6*]

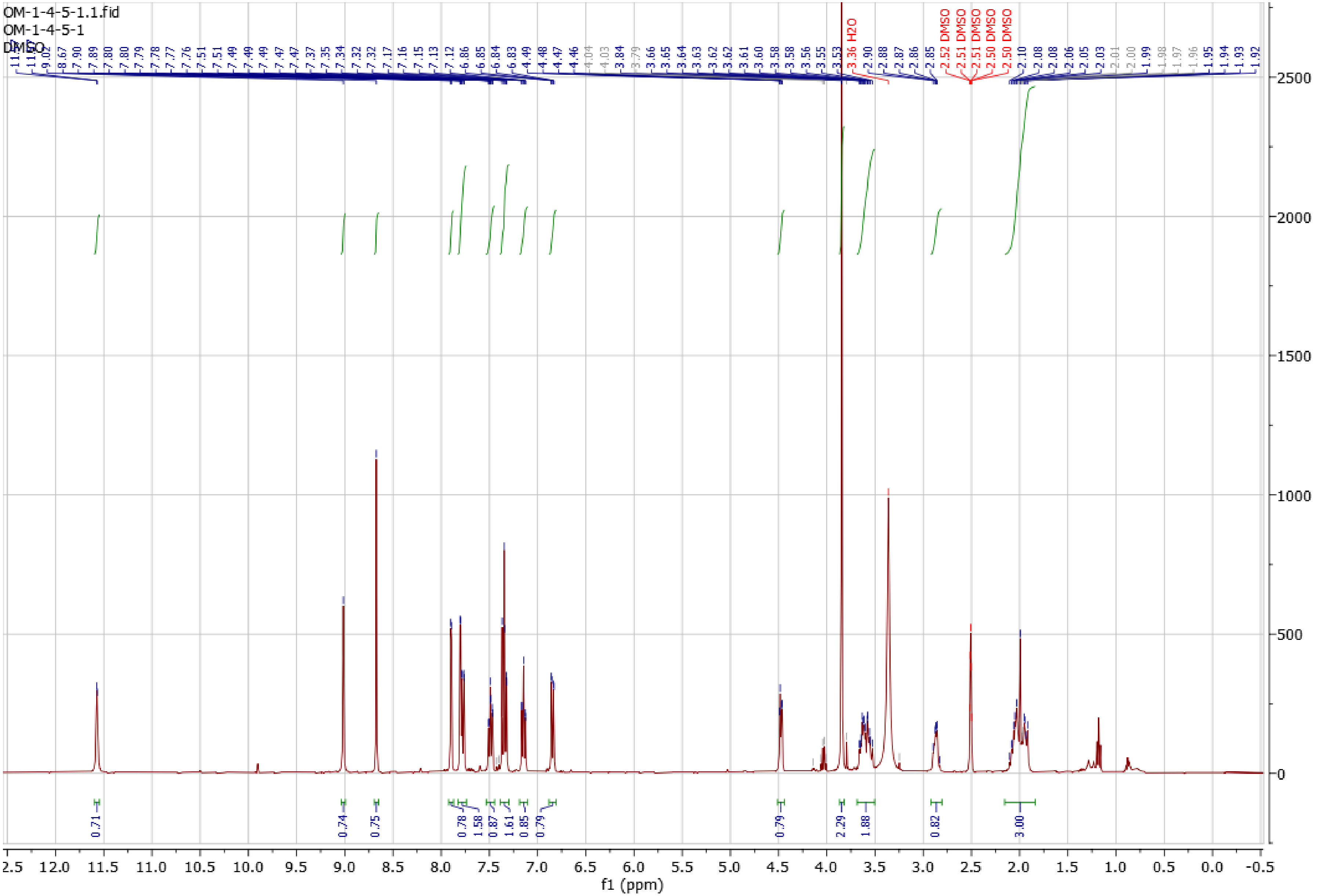

**SI31:** $^{1}$H NMR spectrum of 4p [400 MHz, DMSO-*d6*]

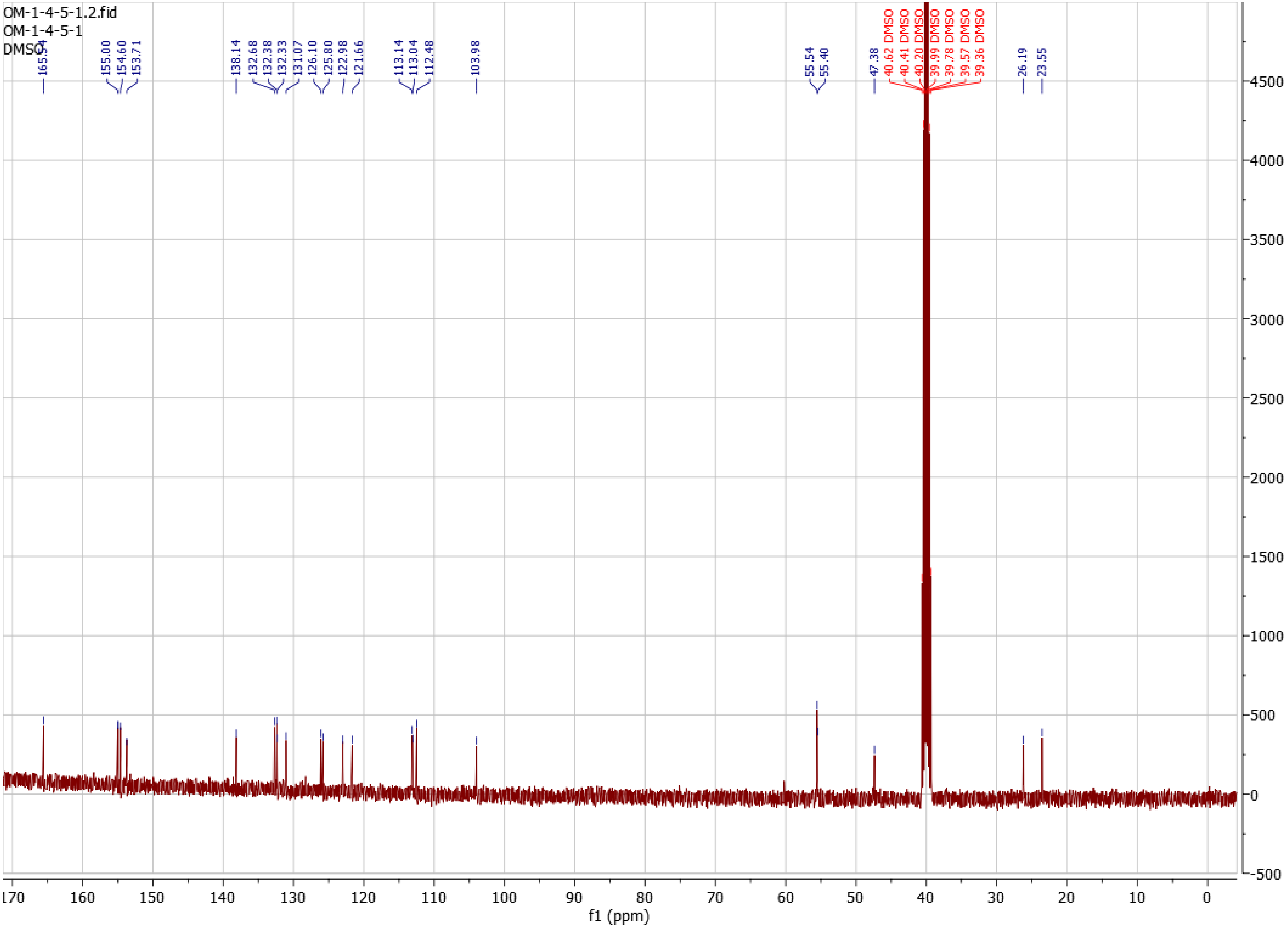


**SI32:** $^{13}$C NMR spectrum of 4p [100 MHz, DMSO-*d6*]

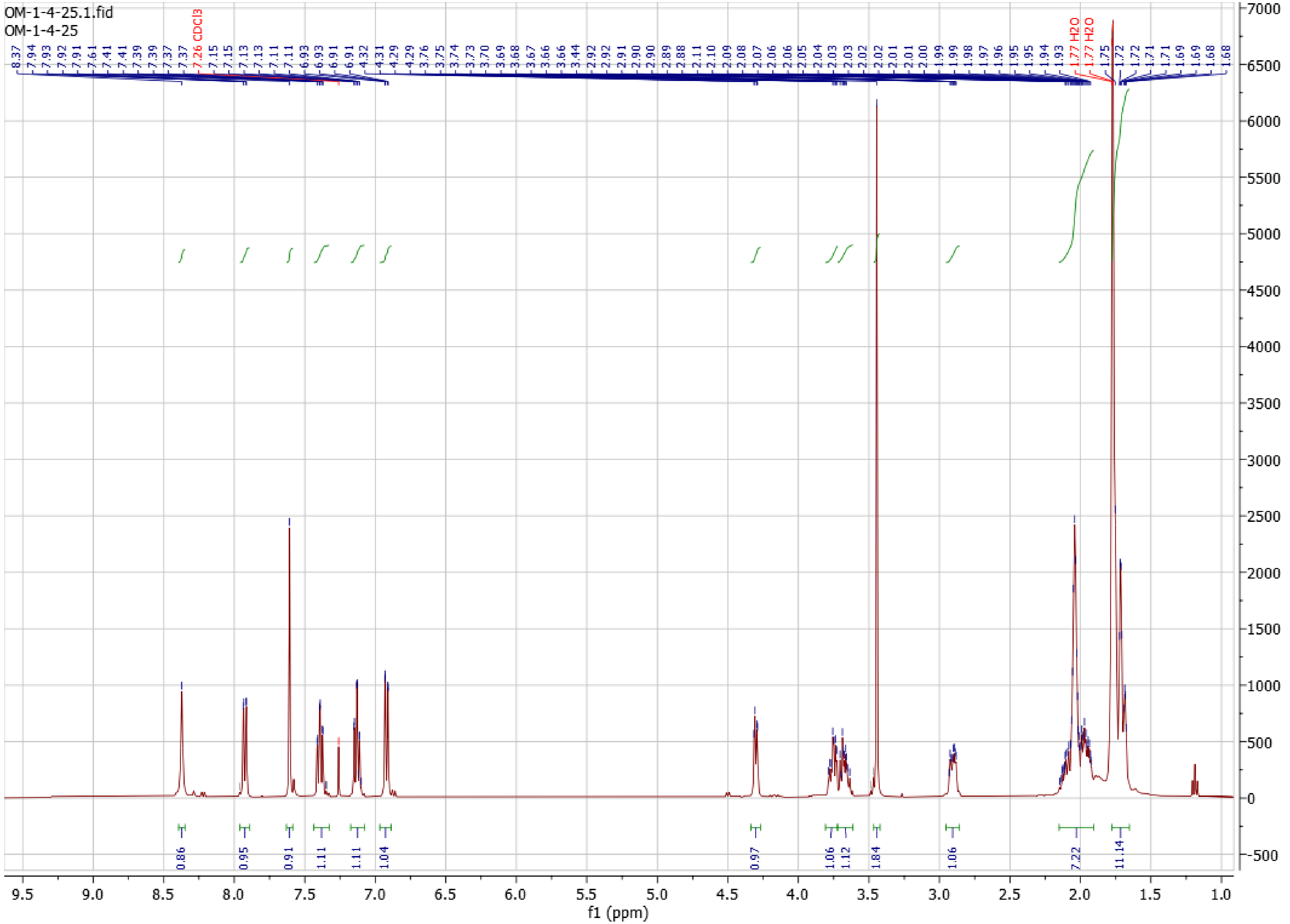

**SI33:** $^{1}$H NMR spectrum of 4q [400 MHz, DMSO-*d6*]

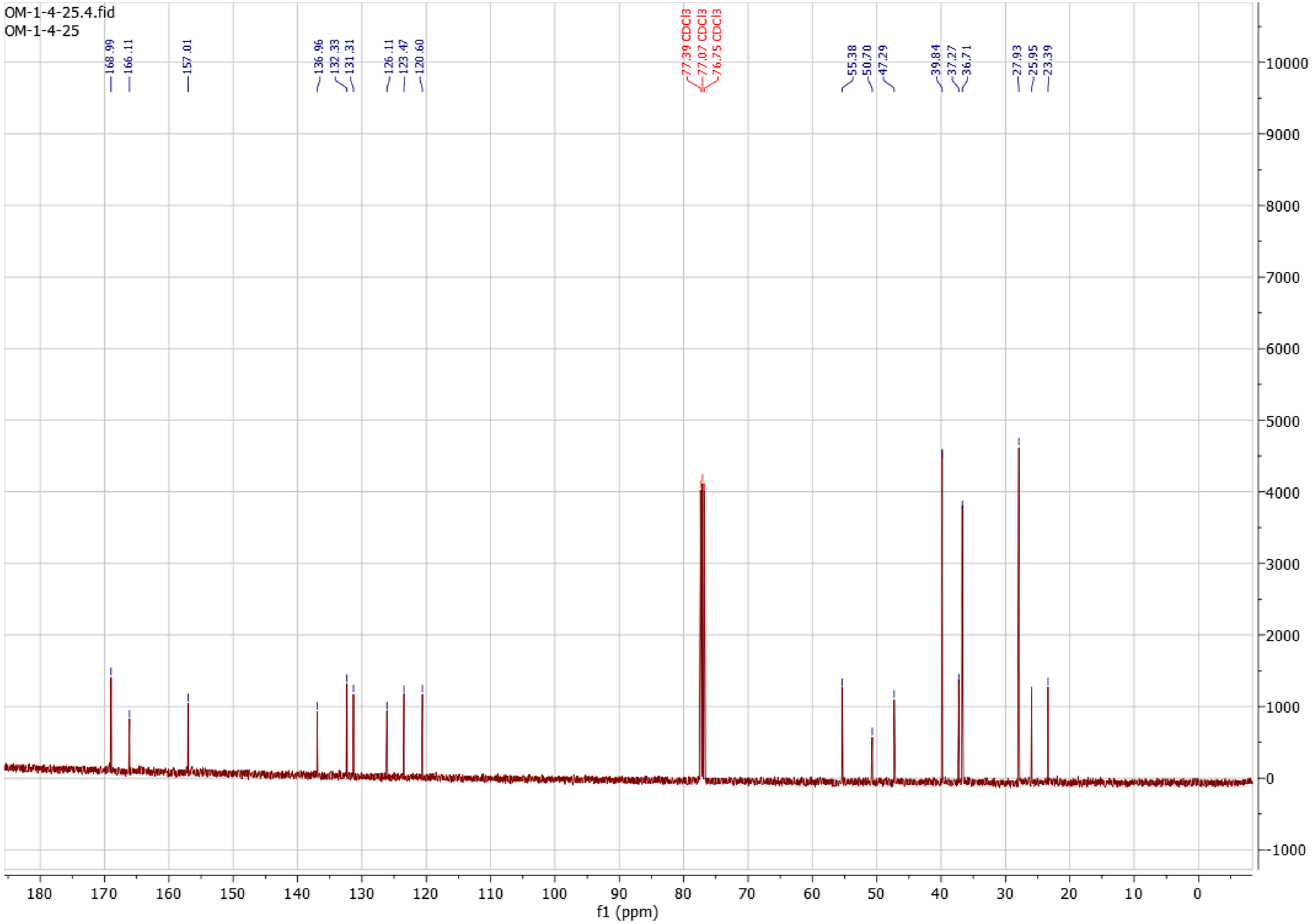


**SI34:** $^{13}$C NMR spectrum of 4q [100 MHz, DMSO-*d6*]

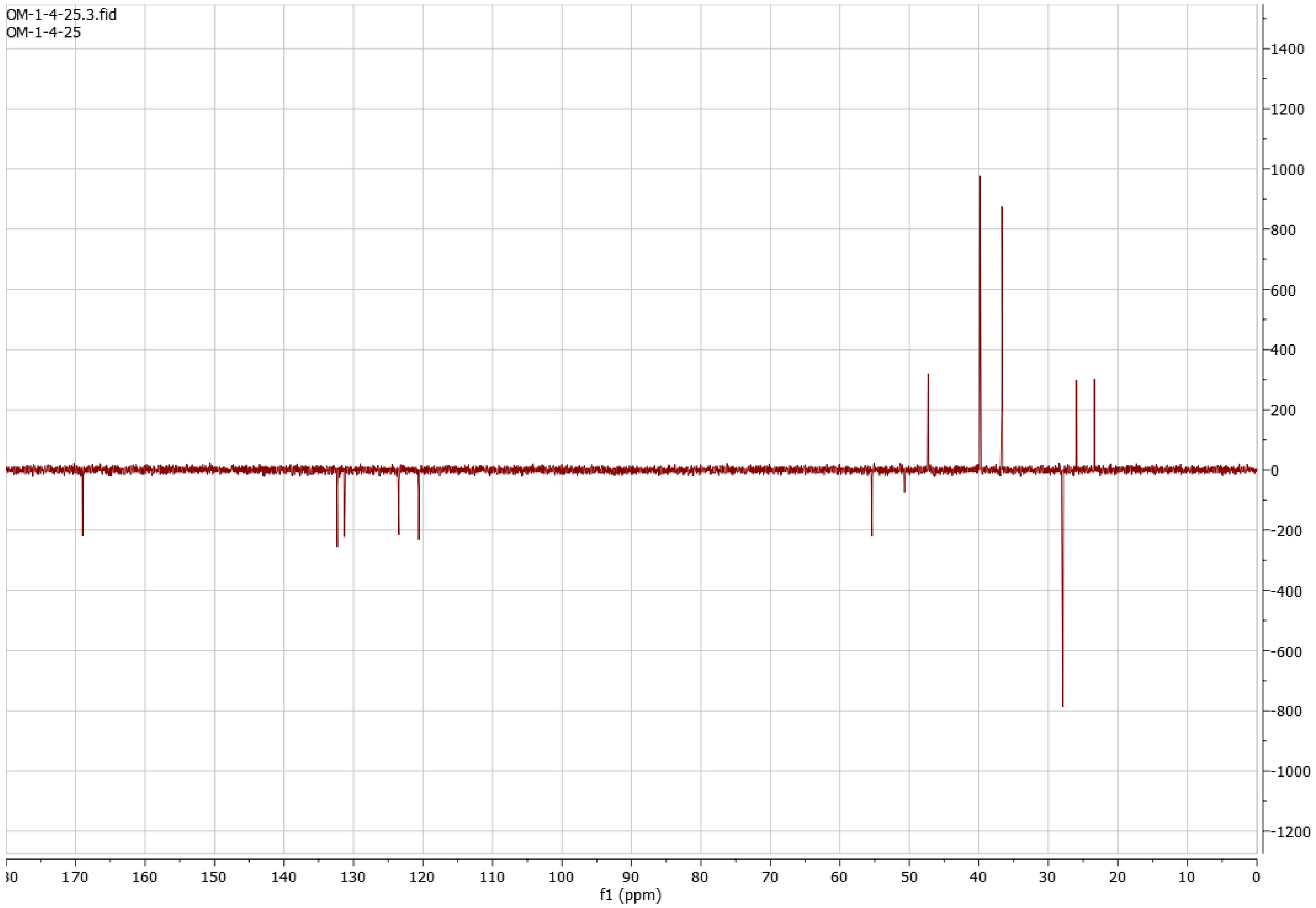


**SI35:** Dept-135 spectrum of 4q [100 MHz, DMSO-*d6*]

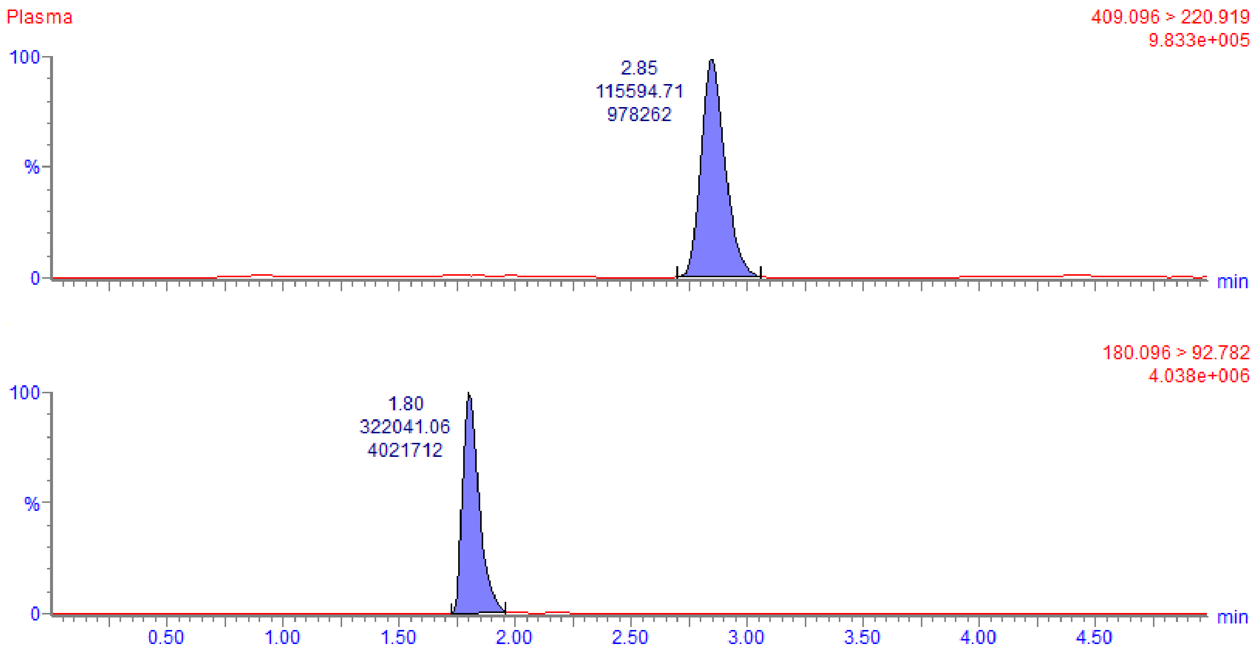


**SI36:** Typical MRM chromatograms of **4k** (top panel) and the IS (below panel) at 0.5 h plasma pharmacokinetic study sample

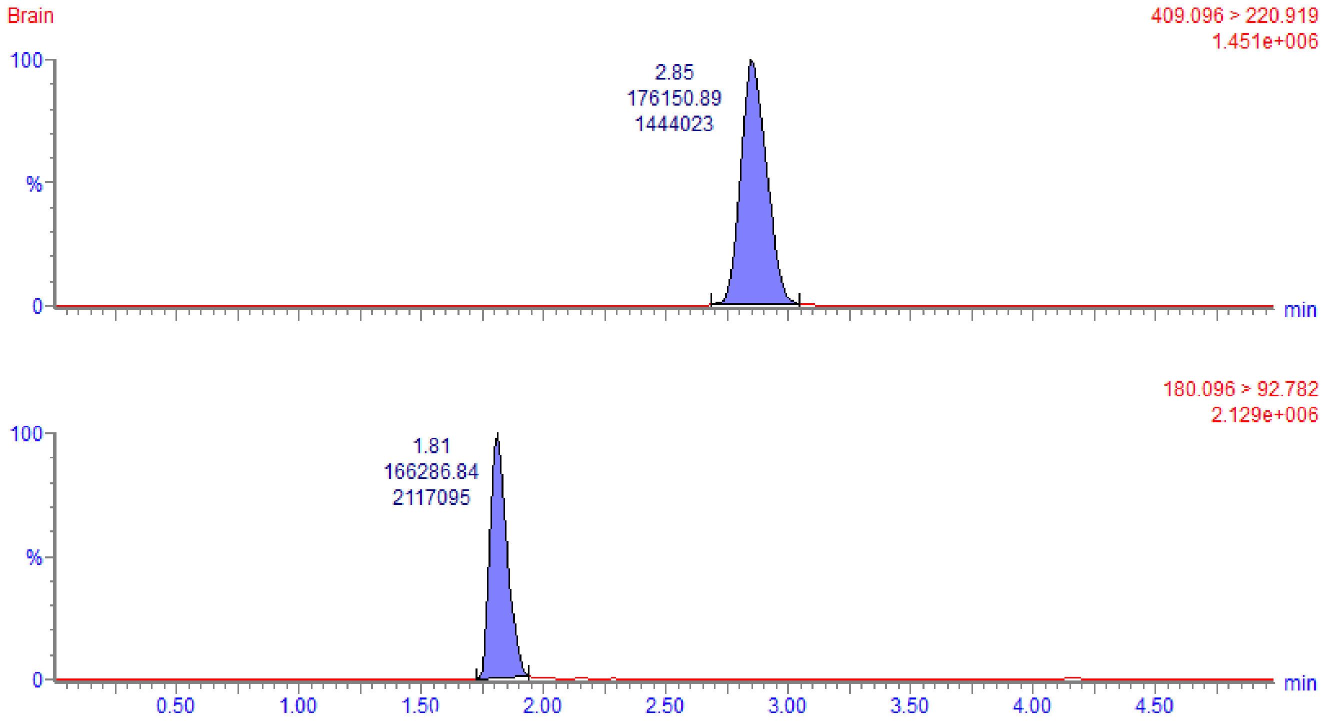


**SI37:** Typical MRM chromatograms of **4k** (top panel) and the IS (below panel) at 0.5 h brain pharmacokinetic study sample

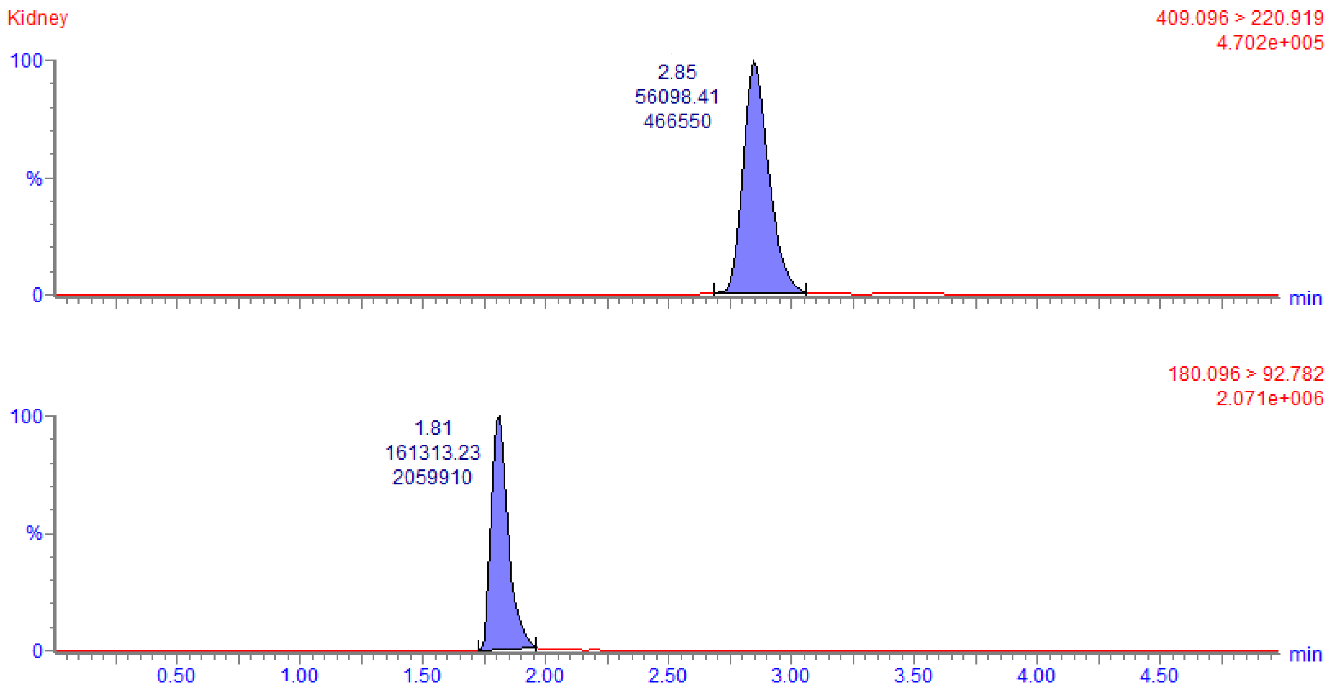


**SI38:** Typical MRM chromatograms of **4k** (top panel) and the IS (below panel) at 0.5 h kidney pharmacokinetic study sample

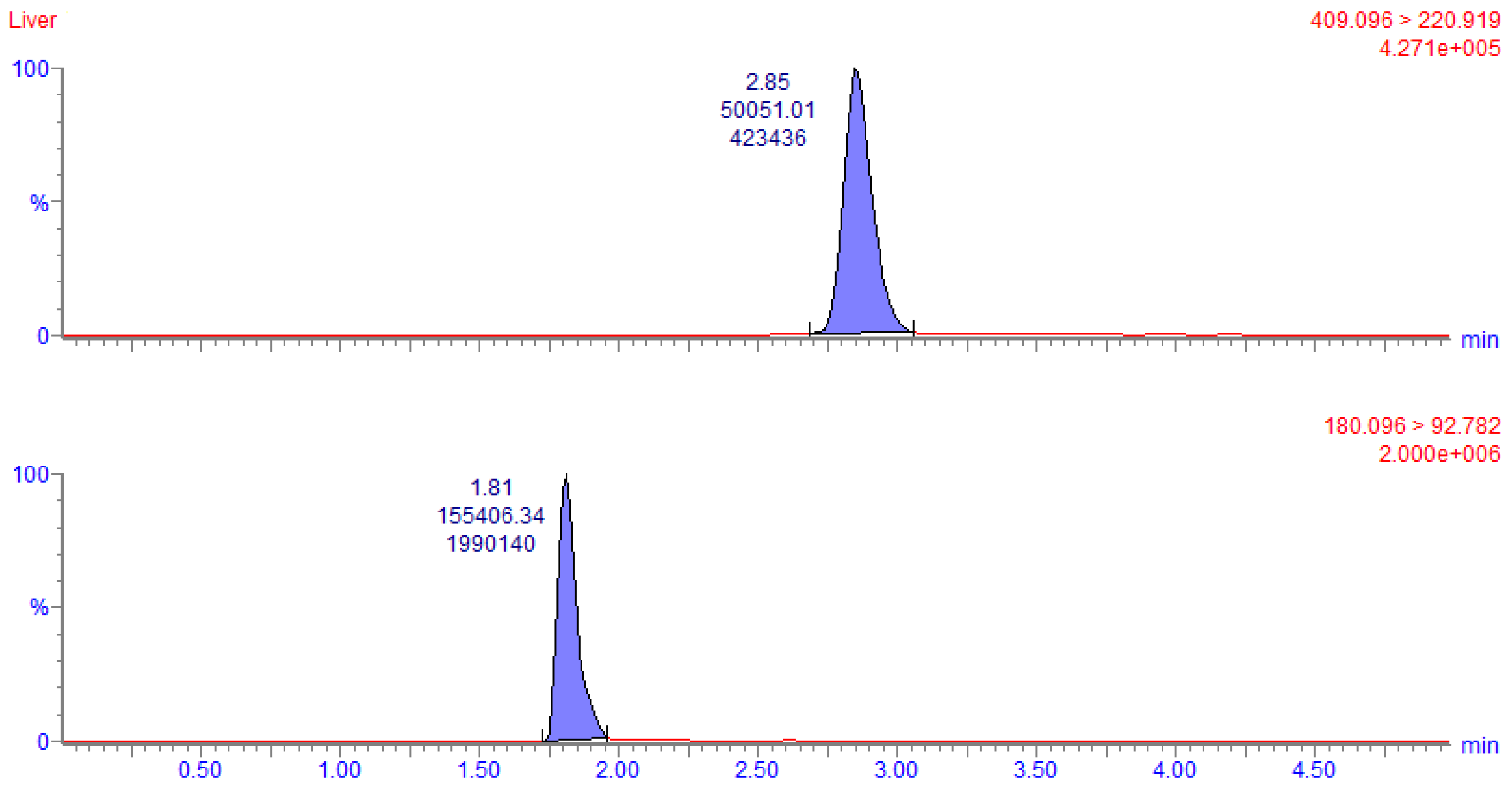


**SI39:** Typical MRM chromatograms of **4k** (top panel) and the IS (below panel) at 0.5 h liver pharmacokinetic study sample
